# Mutational pathway maps and founder effects define the within-host spectrum of hepatitis C virus mutants resistant to drugs

**DOI:** 10.1371/journal.ppat.1007701

**Published:** 2019-04-01

**Authors:** Rubesh Raja, Aditya Pareek, Kapil Newar, Narendra M. Dixit

**Affiliations:** 1 Department of Chemical Engineering, Indian Institute of Science, Bangalore, India; 2 Centre for Biosystems Science and Engineering, Indian Institute of Science, Bangalore, India; Plymouth University, UNITED KINGDOM

## Abstract

Knowledge of the within-host frequencies of resistance-associated amino acid variants (RAVs) is important to the identification of optimal drug combinations for the treatment of hepatitis C virus (HCV) infection. Multiple RAVs may exist in infected individuals, often below detection limits, at any resistance locus, defining the diversity of accessible resistance pathways. We developed a multiscale mathematical model to estimate the pre-treatment frequencies of the entire spectrum of mutants at chosen loci. Using a codon-level description of amino acids, we performed stochastic simulations of intracellular dynamics with every possible nucleotide variant as the infecting strain and estimated the relative infectivity of each variant and the resulting distribution of variants produced. We employed these quantities in a deterministic multi-strain model of extracellular dynamics and estimated mutant frequencies. Our predictions captured database frequencies of the RAV R155K, resistant to NS3/4A protease inhibitors, presenting a successful test of our formalism. We found that mutational pathway maps, interconnecting all viable mutants, and strong founder effects determined the mutant spectrum. The spectra were vastly different for HCV genotypes 1a and 1b, underlying their differential responses to drugs. Using a fitness landscape determined recently, we estimated that 13 amino acid variants, encoded by 44 codons, exist at the residue 93 of the NS5A protein, illustrating the massive diversity of accessible resistance pathways at specific loci. Accounting for this diversity, which our model enables, would help optimize drug combinations. Our model may be applied to describe the within-host evolution of other flaviviruses and inform vaccine design strategies.

## Author summary

The spectrum of viral mutants that exists in infected individuals defines the diversity of drug resistance pathways accessible to any virus. Drug combinations that block these pathways the most effectively are likely to elicit the best responses. The mutants may lie below detection, rendering treatment optimization difficult. We constructed a multiscale mathematical model to estimate the pre-treatment frequencies of the entire spectrum of hepatitis C virus mutants at specific resistance loci. We described intracellular evolution stochastically and extracellular dynamics deterministically, gaining accuracy without escalating computational costs. Model predictions quantitatively captured experimental observations, explained confounding inter-subtype differences, and unraveled the massive diversity of accessible resistance pathways. Our study would help describe viral evolution more accurately, optimize drug treatments and design vaccines.

## Introduction

Direct acting antiviral agents (DAAs) have revolutionized the treatment of chronic hepatitis C virus (HCV) infection, eliciting nearly 100% cure rates in clinical trials with oral treatments often lasting as short as 8 weeks [1]. Efforts are now focused on identifying DAA combinations that prevent the development of drug resistance more effectively and can reduce treatment durations further [2–8]. Mutations that confer resistance to individual DAAs, termed resistance-associated amino acid variants (RAVs), have been identified [9]. The frequencies with which RAVs are likely to exist in individuals before treatment are important to the identification of optimal DAA combinations; DAAs must effectively block the growth of these pre-existing drug resistant strains during treatment [10–12]. Triple-DAA combinations were found recently to lower the likelihood of the development of resistance significantly compared to double-DAA combinations [5]. Current assays are inadequately equipped to estimate the frequencies of minority strains. The assays can detect mutants with frequencies up to ~0.1% [13, 14]. With typical baseline viral loads of 10^6^ copies/ml in chronic infection [15], a mutant frequency of 0.01% would imply ~100 mutant copies/ml, which would go undetected but can be sufficient to cause treatment failure. Indeed, a recent study has argued, using phylogenetic analysis, that resistance to a new DAA observed in a longitudinal study was due to undetected pre-existing RAVs [16]. Mathematical modelling may provide an alternative route to estimating the frequencies of such minority variants and aid the identification of optimal DAA combinations.

Mathematical models have played a crucial role in describing hepatitis C viral kinetics and drug action and have guided treatments [17]. Following the advent of DAAs, the models have been extended to describe the development of drug resistance and to define optimal drug combinations [5, 18–20]. The models, however, are adaptations of models of HIV dynamics [21, 22] and therefore present approximate descriptions of HCV evolution and DAA treatments. Two key challenges must be overcome to develop an accurate model of within-host HCV evolution and estimate the pre-existing frequencies of RAVs.

First, HCV evolution is a multiscale phenomenon, with selection both at the intracellular and extracellular levels. This represents a departure from HIV evolution: An HIV infected cell typically carries a single integrated provirus and produces identical virions [23]. Selection therefore occurs largely at the extracellular level. In contrast, HCV undergoes continuous replication, mutation, and selection within each infected cell [24–26], resulting in potentially diverse progeny virions from each infected cell. Further, each infected cell carries a few hundred HCV RNA copies [27], which makes this evolutionary process strongly stochastic. Finally, infected cells have short lifespans (a few days [28]), which may not allow intracellular evolution to achieve a steady state. Mutation-selection balance, which underlies most current models [18, 21], where the frequency of resistant strains is determined by the balance between mutation of the wild-type yielding the mutant and selection against the wild-type eliminating it, is thus unlikely to hold and founder effects may dominate. Extracellular dynamics, however, is expected to be like HIV, captured by current HCV kinetics models [18, 29–31]. Accurate integration of intracellular and extracellular evolution has been an outstanding challenge [16, 25].

Second, although the positions where mutations confer resistance to DAAs are well defined, the mutations at those positions are not unique [9, 12]. For instance, at the position 155 on the NS3 gene, any of the mutations R155K/I/G/M/T/Q/C/W/N could confer resistance to several NS3/4A protease inhibitors, namely, boceprevir, telaprevir, simeprevir, asunaprevir, paritaprevir, grazoprevir, glecaprevir, and voxilaprevir [9, 32]. An entire spectrum of mutations at the R155 position, thus, can lead to treatment failure, with each mutation representing a potentially independent resistance pathway. Similarly, the mutations Y93H/C/N/R/W/S/T all lead to resistance to the NS5A inhibitors daclatasvir, ledipasvir, ombitasvir, elbasvir, velpatasvir, and pibrentasvir [9, 32]. While R155K is often detected pre-treatment, the other RAVs at this position are not [33]. Accurate estimation of the likelihood of the development of resistance to different DAAs would require quantification of the frequencies of the entire spectrum of RAVs that may exist in a chronically infected individual. Current models have not been designed for this; they are restricted to either the most prominent or the fittest few RAVs or lump all the RAVs into a combined mutant species [18–20].

Here, we constructed a model that overcame both these challenges. Our model could thus estimate the frequencies of the entire spectrum of variants at chosen loci, defining accessible resistance pathways and presenting a framework for the comparative evaluation of DAA combinations.

## Results

### Multiscale semi-stochastic model of within-host HCV evolution

**Fig 1:**
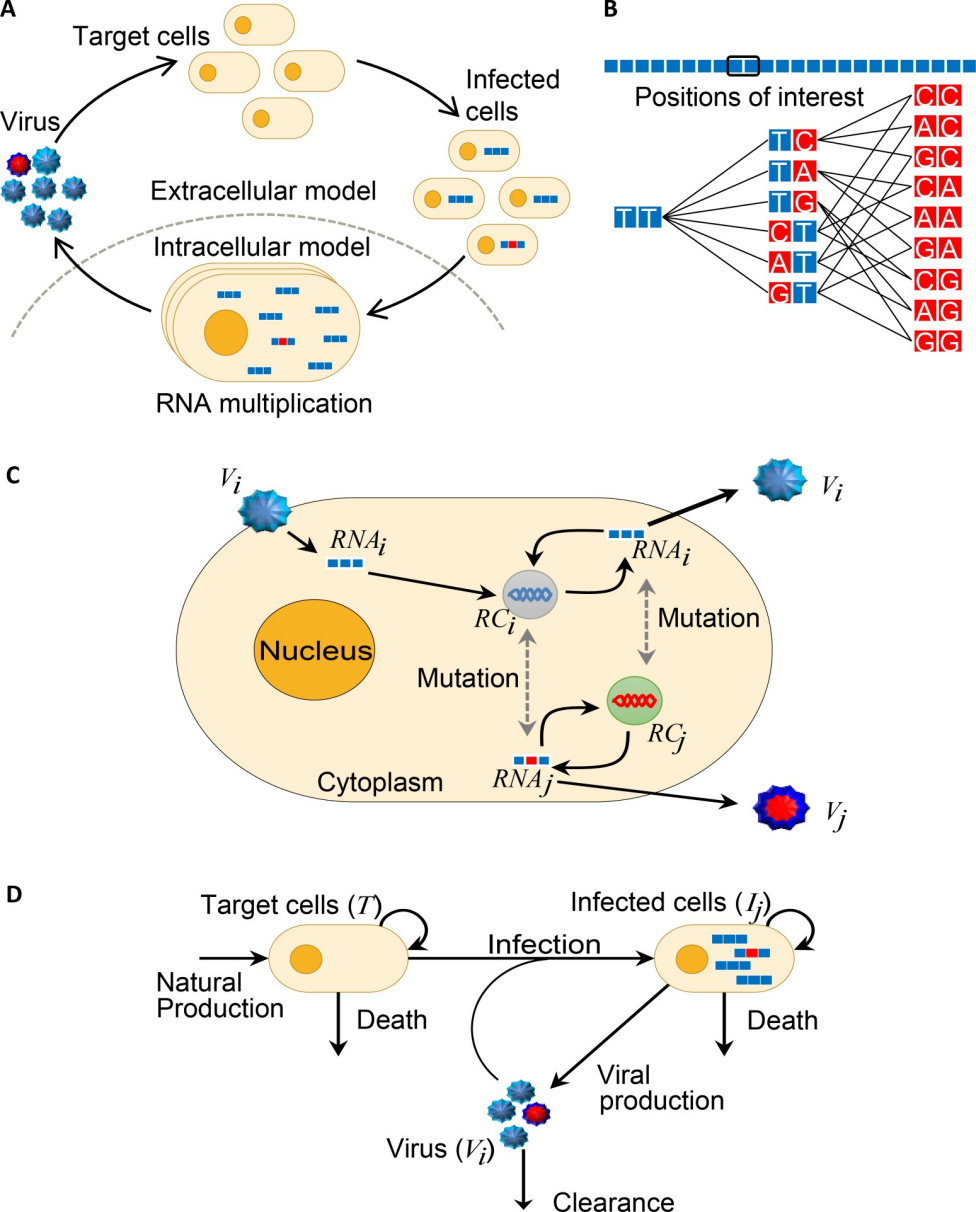
Schematic of the model. **(A)** The overall model architecture demonstrating the infection of target cells by virions to yield infected cells, within which viral replication results in the production of wild-type (blue) and mutant (red) genomes, leading in turn to the production of virions carrying these genomes. The separation into intracellular and extracellular evolutionary and dynamical scales is highlighted. **(B)** Representation of genomes as strings of nucleotides. A hypothetical substring of two nucleotides of interest yields 6 different single mutants and 9 double mutants. **(C)** Schematic of the intracellular model. A virion carrying a genome of type *i* infects the cell, triggering the replication of genomes from positive-strand (*RNA*_*i*_) to negative-strand (*RC*_*i*_) and vice versa. Mutation can give rise to altered genomes, *RNA*_*j*_ and *RC*_*j*_, resulting in the production of virions *V*_*j*_ in addition to *V*_*i*_. The events are summarized along with their rates in Table 2. **(D)** Schematic of the extracellular model. Target cells, *T*, are produced, die, proliferate, and get infected by virions *V*_*j*_ to yield infected cells *I*_*j*_, which also proliferate, produce progeny virions, and die. The probability of infection and the type of virions produced are determined from the stochastic intracellular model. The parameters used are in Table 1.

We constructed a multiscale model of HCV kinetics with stochastic intracellular viral replication and evolution coupled with deterministic extracellular population dynamics (Fig 1A). We represented the viral genome as a string of nucleotides (Fig 1B). We restricted the string to loci where mutations can give rise to resistance to a DAA. We considered genomes carrying all possible mutations at these loci. For instance, for a hypothetical string of two loci, 6 genomes carrying single mutations and 9 carrying double mutations were possible (Fig 1B), all of which were considered in our model. When a single codon associated with resistance to a DAA was considered, a total of 4^3^−1 = 63 different genomes carrying different single, double, and triple mutations became possible. Virions carrying each of these genomes could exist in the viral population in an infected individual. The distribution of these genomes in the population would define the spectrum of mutations at the locus. We quantified this spectrum as follows (see Methods for details). We first performed stochastic simulations of intracellular evolution with each one of the possible genomes as the infecting strain and estimated the probability that the strain established productive infection and, when it did, the distribution of different genomes in progeny virions. Performing a million realizations with every infecting strain, we estimated the mean relative infectivity, *λ*_*j*_, of each strain *j* and the specific release rate, *p*_*ij*_, of virions containing genomes *i* from cells infected with strain *j* for all combinations of *i* and *j*. The simulations involved replication of positive- to negative-strand RNA and vice versa, mutations, distinguished into transitions and transversions, fitness selection, and progeny virion production. The quantities *λ*_*j*_ and *p*_*ij*_ provided inputs to our deterministic model of extracellular dynamics. These quantities modified the standard model of viral kinetics by accounting for the effects of mutations on viral infectivity and the distribution of genomes in progeny virions. Solving the resulting equations, using parameters representative of HCV infection in vivo (Table 1), we obtained the within-host frequencies of all variants, quantifying the spectrum of mutants at any chosen loci.

**Table 1:** Model parameters and their values.

	Symbol	Description	Value	Source
Intracellular model	*k*_+_	Replication rate of RNA to RC	0.1 h^-1^	S1 Text
	*k*_−_	Replication rate of RC to RNA	3.0 h^-1^	S1 Text
	K	Carrying capacity of a cell for RNAs and RCs	270	S1 Text
	*f*_*j*_	Fitness landscape	Fig 2B inset; S5 Fig	[19, 38]
	*d*_*RNA*_	Degradation rate of RNA	0.046 h^-1^	[25]
	*d*_*RC*_	Degradation rate of RCs	0.058 h^-1^	[25]
	*μ*_*ts*_	Transition rate	2.7×10^−5^ per site per replication	[38]
	*μ*_*tv*_	Transversion rate	1.5×10^−6^ per site per replication	[38]
	*ρ*	Rate of release of virions from an infected cell	0.01 virions h^-1^	[25]
Extracellular model	*s*_*gen*_	Generation rate of target cells	*d*_*T*_*K*_*cell*_	[28]
	*β*	Infection rate constant	10^−7^ ml day^-1^ virion^-1^	[18]
	*d*_*T*_	Death rate of target cells	0.004 (0.001–0.014) day^-1^	[28]
	*k*_*prt*_	Proliferation rate of target cells	1.25 (1–3) day^-1^	[28]
	*k*_*pri*_	Proliferation rate of infected cells	0.125 day^-1^	[28]
	*K*_*cell*_	Carrying capacity of the liver for cells	1.3×10^7^ cells ml^-1^	[18]
	*N*	Number of non-target cells	*K*_*cell*_/2	[18]
	*δ*	Death rate of infected cells	0.33 (*d*_*T*_−0.5) day^-1^	[28]
	*c*	Clearance rate of free virions	4.2 (0.8–22) day^-1^	[28]

### Intracellular dynamics and patterns of evolution

**Fig 2:**
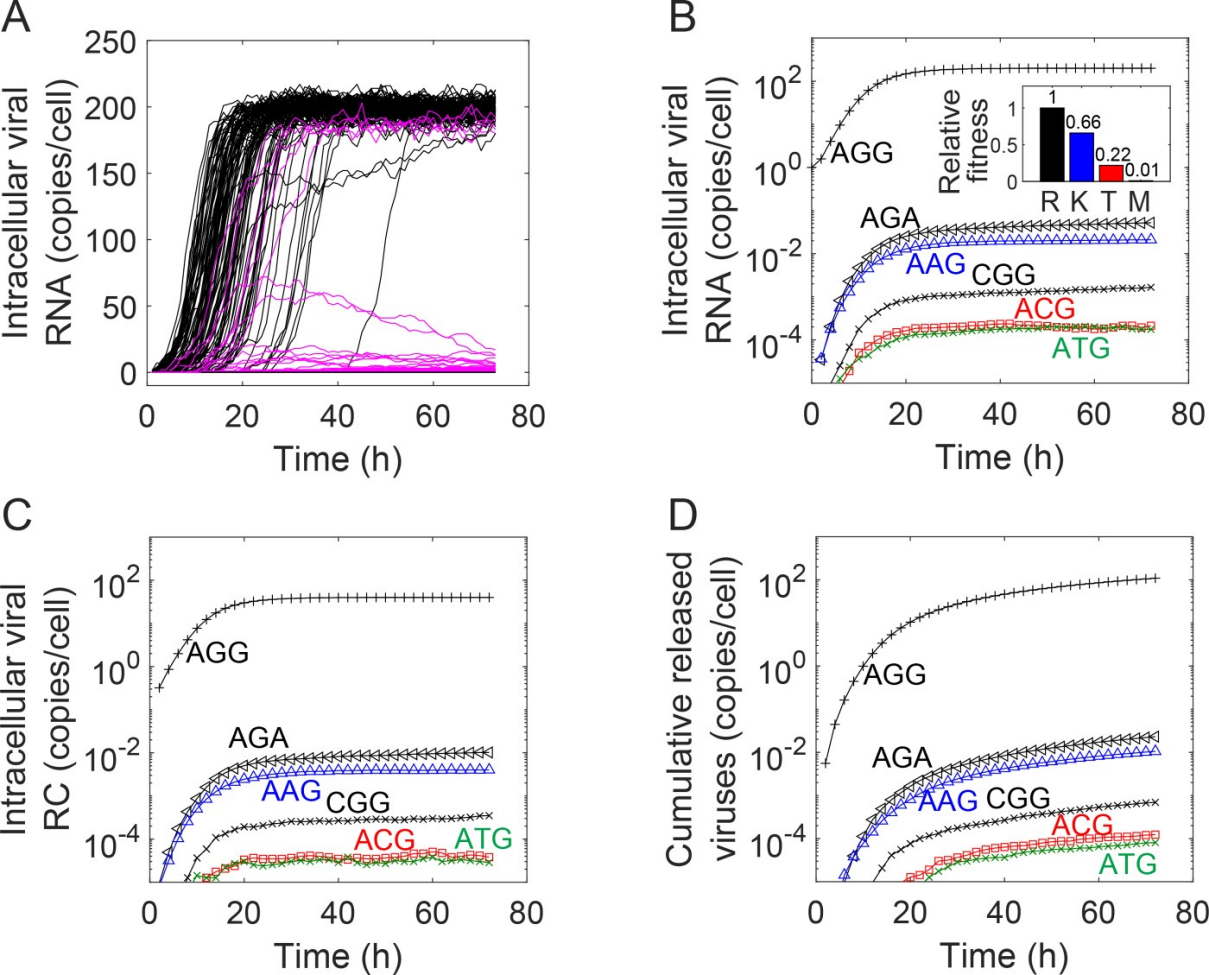
Intracellular dynamics and evolution. **(A)** Time-evolution of the populations of wild-type (black) and single mutant (pink) RNA genomes in infected cells. Infection is initiated by the wild-type. Each trajectory is a realization. The three patterns where the wild-type dominates, the mutant dominates, and where the mutant rises initially but is eventually outcompeted are illustrated. **(B)** The averaged evolution of the populations of genomes carrying different codons following infection with AGG. The relative fitness of the genomes, determined independently [19], is in the inset and is color coded. **(C), (D)** The corresponding populations of replication complexes and virions released.

We first considered the position 155 in the NS3 protease of HCV, where mutations yield resistance to NS3/4A protease inhibitors, such as telaprevir [9, 12]. The wild-type HCV genotype 1a contains the amino acid arginine (R) represented by the codon AGG at this position [12]. We performed stochastic simulations of intracellular evolution with the infecting strain containing the codon AGG. Mutations could yield different amino acids, such as lysine (K) and threonine (T). The relative fitness of the RAVs at this position has been estimated previously; only the RAVs K, T, and methionine (M) had non-zero fitness [19]. We employed these fitness values in our simulations. (These fitness values were found to correlate well with estimates from in vitro studies [19, 34]. Using the latter in vitro values, which were available for a wider set of RAVs, made the computations more complex because of the presence of the additional mutational pathways, but did not change our estimates significantly (S1 Fig).) We examined individual realizations and found that in most realizations the population of the infecting genome rose from one to nearly the carrying capacity of the cell, where it stabilized (Fig 2A). Other genomes were rarely present. The time when the population began to rise, indicating the onset of viral replication, varied significantly across cells, with some cells seeing the rise soon after infection whereas others seeing it as late as 40 h after infection. The initiation of replication was thus subject to strong stochastic fluctuations. If the infecting genome were to be degraded before the initiation of replication, the cell would cease to be productively infected (see below).

In some realizations, where the infecting genome experienced a mutation early on, the population came to be dominated by the mutant, which reached the carrying capacity and stabilized. In a small minority of realizations, where the mutant population was on the rise, a reverse mutation leading to the infecting genome occurred. The infecting genome then grew at the expense of the mutant because of its higher relative fitness. Eventually, the infecting genome came to dominate the population and the mutant died down, a pattern akin to the replacement of a less fit strain following superinfection with a fitter strain [35].

Thus, three patterns of intracellular evolution were evident (Fig 2A). The first, which occurred in a vast majority of the realizations, was where the infecting genome dominated the population; the second, which occurred in a minority, was where the mutant dominated; and the third, which occurred in a smaller minority, was where the mutant dominated initially but was eventually outcompeted by the infecting genome.

### Founder effects

We examined next the average evolution across a large number (10^6^) of realizations. We found that the intracellular population was dominated by the infecting strain, which existed at levels close to the carrying capacity of ~200 genomes per cell (Fig 2B). The mutants were present in a small minority, ranging on average from 10^−1^ to 10^−4^ genomes per cell; *i*.*e*., one mutant-dominated cell in 10 to 10000 infected cells. The types of mutants present and their frequencies again indicated strong founder effects. All the mutants present were single mutants; double and triple mutants were hardly observed. Further, even the mutations that were synonymous, such as AGA, which did not lead to a fitness penalty, were present in extremely small numbers. This implied that mutations occurred rarely, as expected [25], and cells predominantly carried viral genomes of the type that infected them. Simulations with a two-locus/two-allele model, which were simpler but easier to visualize, corroborated these results (S2 Fig).

### Transition-transversion bias

For the infecting strain AGG, five single mutants with non-zero fitness were possible: CGG, AGA, AAG, ACG, and ATG. Of these, CGG and AGA were synonymous–encoding R–and so introduced no fitness penalty. Yet, they were present at different frequencies, with CGG several orders of magnitude lower than AGA (Fig 2B). This was because CGG required a transversion from AGG, whereas AGA could be produced by a transition. The higher probability with which the latter could be produced thus resulted in the different frequencies. The other three single mutants encoded the amino acids K, T, and M, respectively, which had fitness decreasing in that order (Fig 2B inset). Further AAG required a transition, whereas ACG and ATG required transversions. Thus, AAG was present in higher frequencies than the other two. It was also present at a higher frequency than CGG, which had a higher fitness but required a transversion. CGG, however, was present at a frequency higher than ACG and ATG, the latter present at similarly low frequencies, dictated by their low fitness and the low transversion rate.

The distribution of replication complexes too followed the same trends, with the wild-type dominant and single mutants alone present in small minorities with the ordering of the mutant frequencies defined by the relative fitness and whether a transition or transversion to the infecting strain was required (Fig 2C). Accordingly, the progeny virions released were also predominantly of the type that contained the wild-type genomes (Fig 2D). This transition-transversion bias is consistent with previous studies [36].

Together, these findings implied that strong stochastic and founder effects resulted in the dominance of the infecting strain within cells. The mutation-selection balance, often invoked to describe the frequencies of mutant strains [18, 21], did not hold. Had the mutation-selection balance been achieved, the population would have been dominated by the fittest strain, the wild-type, regardless of the infecting strain. The small intracellular carrying capacity, the low mutation rate, and the short lifespan of infected cells together precluded the mutation-selection balance from being established.

### Relative infectivity and specific release rate

**Fig 3:**
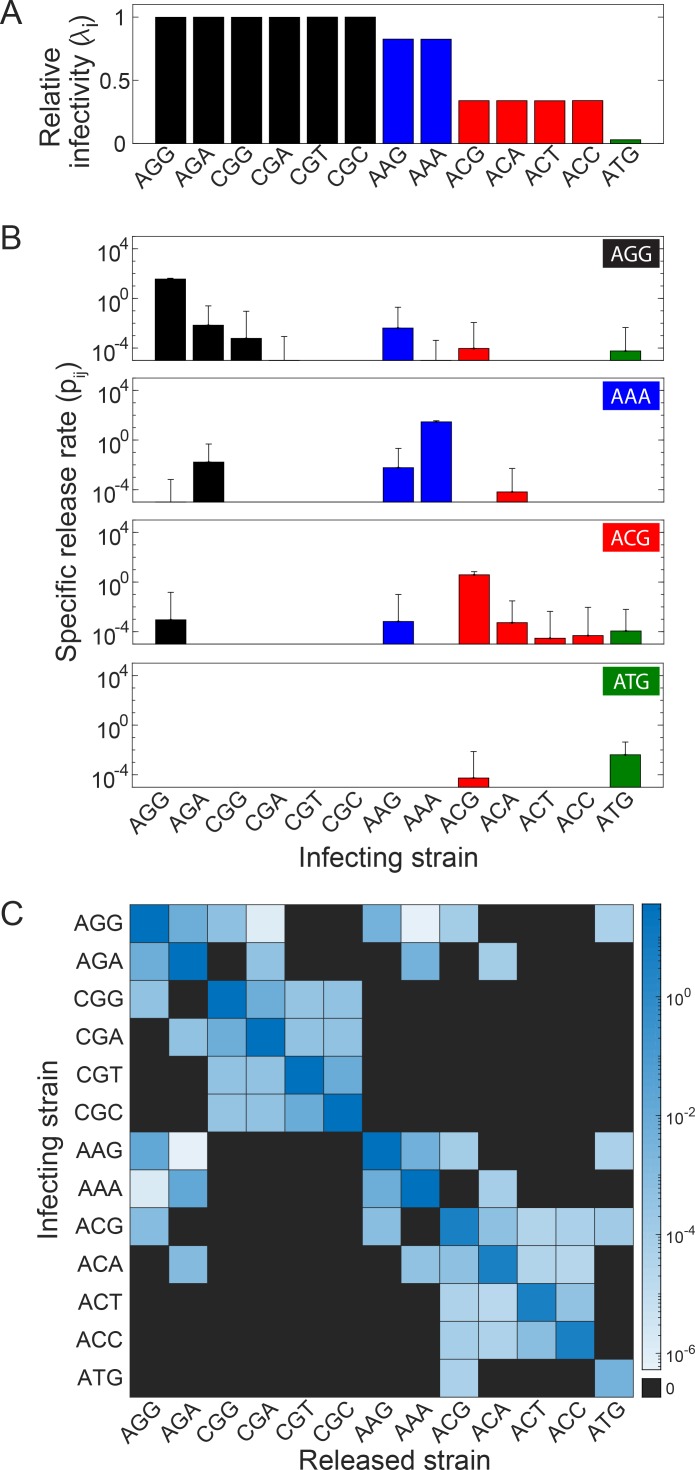
Relative infectivity and specific release rate. **(A)** Relative infectivity of strains carrying different codons obtained from stochastic simulations. The codons are color coded by the amino acids they encode (see inset of Fig 2B). **(B)** The average rate at which virions carrying different codons are released following infection with a virion carrying particular codons mentioned in the panels. Estimates for the remaining infecting strains are in S3 Fig **(C)** A heat map displaying the specific release rate estimated in (B) and S3 Fig compactly. The actual values are in S1 Table.

We repeated the simulations above with every strain, 13 in all, that had a non-zero relative fitness as the infecting strain and estimated the relative infectivity, *λ*_*j*_, and the specific release rate, *p*_*ij*_, which provided the necessary inputs to the extracellular model. We found that *λ*_*j*_ was dependent on the amino acid of the infecting strain and not the codon and decreased as the fitness of the infecting strain decreased (Fig 3A).

When productive infection did occur, *p*_*ij*_ increased overall with the fitness of the infecting strain (Fig 3B; S3 Fig; S1 Table). Thus, more virions were produced from an infected cell on average when the infecting strain was AGG than ATG. The virions produced, however, were predominantly of the type that contained the infecting genome regardless of fitness (Fig 3B); *i*.*e*., for any infecting strain *j*, *p*_*jj*_>*p*_*ij*_. For instance, even with ATG as the infecting genome, which had the least relative fitness (Fig 2B inset), the dominant progeny virion type was the one containing ATG (Fig 3B). Further, *p*_*ij*_ dropped to zero for all *i* removed from *j* by more than one mutation; *i*.*e*., no genomes containing more than one mutation in the infecting strain were produced. Finally, *p*_*ij*_ was lower for values of *j* that required a transversion from *i* compared to those that required a transition, reiterating the transition-transversion bias. *p*_*ij*_ (Fig 3B and S3 Fig) are collated in a heat map (Fig 3C).

Using *λ*_*j*_ and *p*_*ij*_ estimated thus, we solved our model of extracellular dynamics.

### Short-term extracellular dynamics and evolution

**Fig 4:**
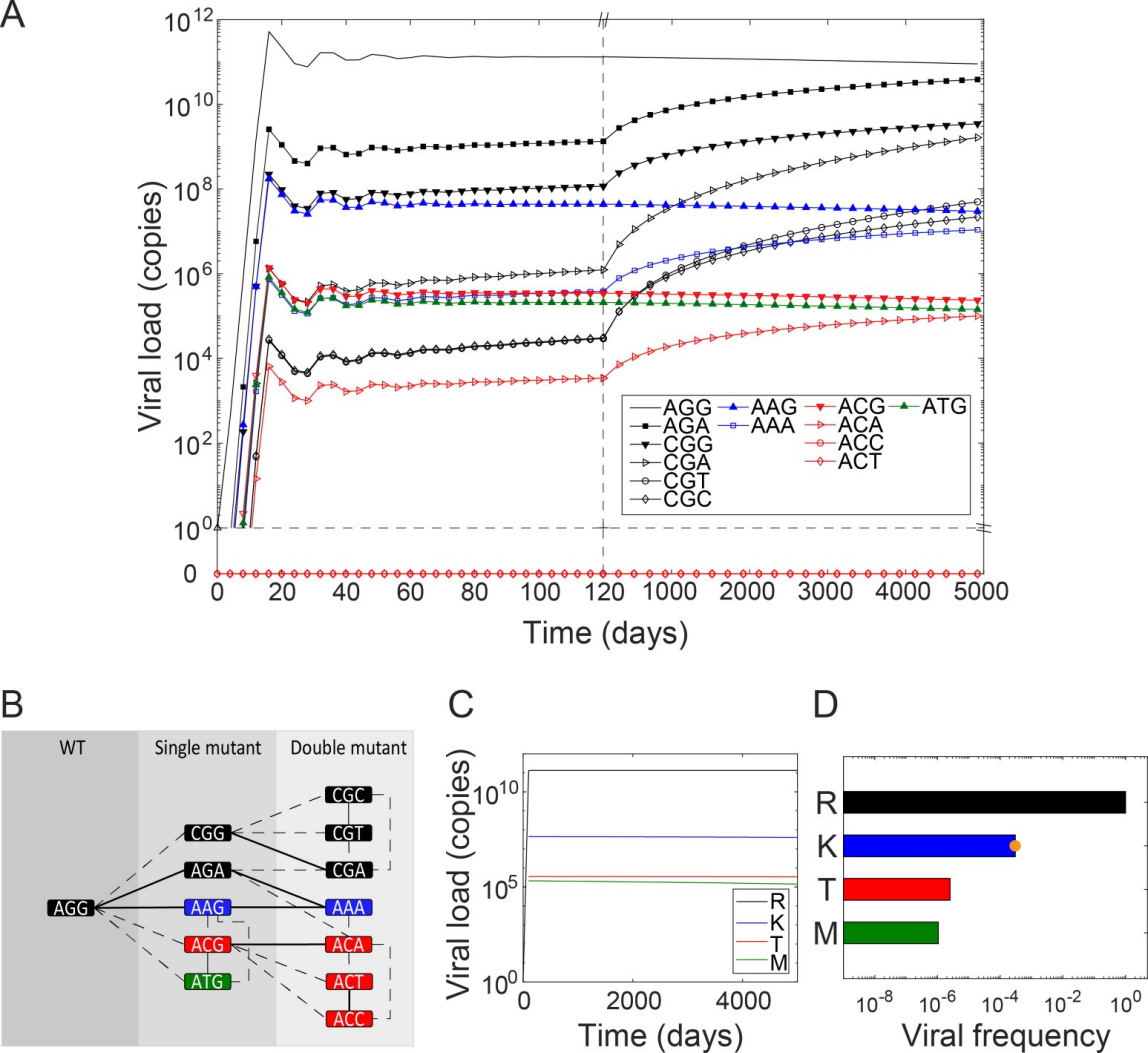
Extracellular dynamics, the mutational pathway map, and the mutant spectrum. **(A)** The time evolution of virions containing the infecting strain (AGG) and each of the possible mutant codons, color coded by the amino acids they encode. **(B)** A map of mutational pathways leading from one codon to another. Transitions are in solid lines and transversions in dashed lines. The lines connect codons separated by a single mutation. The shades of grey indicate the number of mutations from the infecting strain, AGG. **(C)** The time evolution of the populations of virions grouped by the amino acid variants they contain. **(D)** Frequencies of the different mutants at steady state. Shown for comparison is the database value of the mutant R155K for HCV genotype 1a (orange dot) [33].

We let infection begin with a founder virion containing the wild-type genome with the codon AGG. The viral population quickly rose and, in a few weeks, reached a set point of approximately 10^11^ virions in the infected individual (Fig 4A), consistent with observed viral loads in chronically infected individuals [15]. The population consisted predominantly of virions containing AGG. 12 different mutants, corresponding to amino acids with non-zero fitness, were also present but in much lower numbers. The mutant numbers ranged from ~10^3^ to ~10^9^ virions in the individual, yielding frequencies of approximately 10^−8^–10^−2^, during the first few months of the infection.

### Mutational pathway map

To understand this wide distribution of mutant frequencies, we constructed a map of mutational pathways (Fig 4B). The map grouped codons separated by the same number of mutations from the wild-type into distinct layers, indicated with increasingly lighter shades of gray. Thus, the single mutants, CGG, AGA, AAG, ACG, and ATG, formed the first layer next to the wild-type. These were all the mutant codons that could be produced from a cell infected with the wild-type. The codons are colored based on the amino acids they encode. They are connected to the wild-type by solid or dashed lines depending on whether the mutation involved is a transition or transversion. Accordingly, codons connected with solid lines were more likely to be produced than those with dashed lines. In the same way, we connected codons in the second layer, the double mutants, with those in the first layer. Codons within a layer were also connected when they were removed from each other by a single mutation. The resulting map provided a complete set of accessible mutational pathways at the locus in consideration. Using the map, we understood the distribution of mutants predicted by our model as follows.

### The mutant spectrum

Strong founder effects at the intracellular level implied that most infected cells would carry and produce virions containing the wild-type. A small fraction of the virions produced would be single mutants, determined by the specific release rate estimated above (Fig 3C). These mutants would in turn infect other cells with probabilities determined by their relative infectivity (Fig 3A). The latter cells would produce virions predominantly containing the respective single mutants. A small percentage of the progeny would yield double mutants, which would in turn infect cells and expand their population. Among the single mutants, the easiest to produce were the ones that required transitions, *viz*., AGA and AAG. Of these, AGA encodes R–it has a synonymous mutation–and therefore was as fit as the wild-type, whereas AAG encodes K and was less fit. Among the mutants, we thus found AGA present in the highest numbers. Next in numbers were CGG and AAG. CGG involved a transversion and was therefore harder to produce than AAG, but was synonymous and therefore fitter than AAG. Well below these numbers were the other single mutants, ACG and ATG, which required transversions and were much less fit.

The ordering of the double mutants can again be understood following the above arguments. CGA had the highest fitness (R). It was also produced by a transition and a transversion from the single mutants CGG and AGA, respectively, which had the highest numbers among single mutants. CGA therefore occurred in the highest numbers among the double mutants. The other double mutants with the highest fitness (encoding R), CGC and CGT, both required transversions from their single mutant parent CGG, and were therefore much less represented than CGA. Much higher than them was the double mutant AAA, which could be produced by transitions from two single mutants, AGA and AAG, the former present in high numbers. Yet, it was less prevalent than CGA because it encoded K and was thus less fit. Of the three other double mutants possible, ACA, ACT and ACC, only the former was observed, in low numbers, because it was produced by a transition from its parent single mutant ACG, which in turn was present in small numbers because of its low fitness. The latter two double mutants, although they were as fit as ACA, were not observed at all (their numbers were below one) because they had to be produced by transversions from ACG, which given the strong founder effects was not realized.

Thus, the spectrum of mutants was determined not by the fitness of the mutants alone, but also by founder effects and the mutational pathways involved. Mutants that could be produced by transitions via multiple pathways involving well represented ancestral mutants were present in significant numbers even if their fitness was low.

### Long-term extracellular dynamics

The long-term dynamics was dictated by fitness effects. Gradually, all the mutants encoding the same amino acid converged to the same frequency. The frequencies were then ordered according to the fitness of the amino acids. This long-term evolution, however, was slow and required many tens of years because of the absence of a fitness difference between the codons encoding the same amino acids (Fig 4A).

### Comparison with experiment

We applied our model to estimate the frequency of the R155K mutant and compared it with experimental observations. No experiments have thus far measured the entire spectrum of mutants at any locus *in vivo*. Measurements in infected individuals sample a few genomes (e.g., [16]), which may leave estimates of frequencies subject to uncertainties. Besides, the frequencies are typically below current assay detection limits. We therefore considered the frequency of mutants in public HCV sequence databases, which we expected to be representative of the frequency in typical HCV infected individuals. From 3328 sequences of HCV genotype 1a in public databases, the position 155 was found to have the wild-type amino acid R in 99.82% of the sequences and the mutant K in 0.03% of the sequences [33]. The study was published in 2008, before the advent of DAAs, so that transmitted drug resistance may be ignored.

To compare with these findings, we grouped our codon distributions into their respective amino acid populations and computed the frequencies. We found that although the populations of genomes carrying different codons encoding the same amino acid were gradually varying (Fig 4A), their total populations had nearly reached steady levels (Fig 4C). We found from these steady populations that the frequency of the R155K mutant was 0.03% (Fig 4D), in excellent agreement with the frequency in the public databases [33], giving us confidence in our model predictions. The other mutants, R155T/M, were present at far lower frequencies of ~0.0001%. Previous models underpredict mutant frequencies (S4 Fig), highlighting the improved accuracy of our model.

We applied our model next to two clinically relevant questions, which also highlighted the novelty, scalability, and the wider applicability of our approach.

### Difference between HCV genotypes 1a and 1b

**Fig 5:**
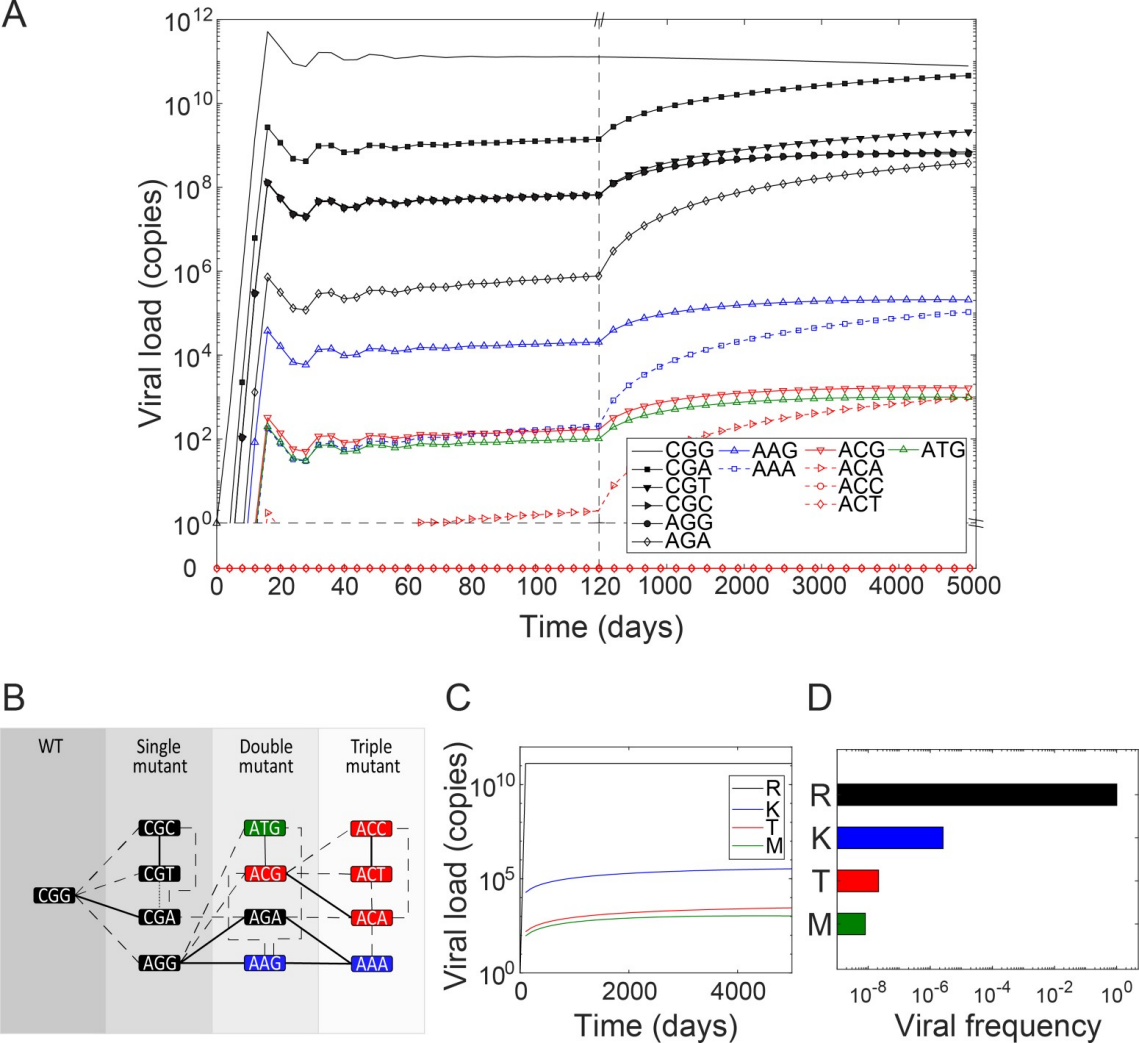
The mutant spectrum for HCV genotype 1b. **(A)** The time-evolution of population of virions carrying different codons, color coded by the amino acids they encode. **(B)** The mutational pathway map with CGG, the wild-type codon for HCV genotype 1b at position 155 of the NS3 protein, as the founder. Transitions are in solid lines and transversions in dashed lines. The lines connect codons separated by a single mutation. The shades of grey indicate the number of mutations from the infecting strain, CGG. **(C)** The time-evolution of variants grouped by amino acids. **(D)** The corresponding frequencies of the different variants.

An intriguing clinical observation has been the significantly lower detection rate of the R155K RAV in HCV genotype 1b infected individuals compared to genotype 1a infected individuals and therefore better responses to NS3/4A inhibitors in the former [10, 12]. To understand this difference, we performed calculations that mimic infection with HCV genotype 1b. The calculations above mimicked genotype 1a infection. We could use the intracellular results above (Fig 3) because the same codons were involved in genotype 1b infection. We assumed that the relative fitness was the same as genotype 1a. The extracellular dynamics had to be recomputed using the appropriate founder strain. The wild-type codon for R in genotype 1b at the position 155 is CGG [12]. The mutational pathway map with this wild-type indicated that no viable single mutants were non-synonymous (Fig 5B). The mutant AAG, which encodes K, required two mutations to CGG. Further, the first of these mutations was a transversion to AGG, which was followed by a transition to AAG. Accordingly, the viral population was not only dominated by the wild-type, the mutant spectrum was dominated by the single mutants which were all synonymous (Fig 5A). The resistant mutant AAG was present in very low numbers, ~10^4^−10^5^ virions, in contrast to the ~10^8^ virions in HCV genotype 1a infection (Figs 4A and 5A). Aggregating the codons into their respective amino acids (Fig 5C), we found that the frequency of the R155K mutant was ~0.0001%, nearly 100-fold lower than the corresponding frequency in the case of genotype 1a (Fig 5D). The other mutants (R155T/M) were present at far lower frequencies. This significantly lower presence of the RAVs in HCV genotype 1b compared to 1a presents a plausible explanation of the less frequent detection of RAVs in individuals infected with the former and may contribute to their better response to NS3/4A inhibitors.

### Multiple pathways of resistance to NS5A inhibitors

To demonstrate the scalability and wider applicability of our model, we considered another class of DAAs, NS5A inhibitors. Unlike NS3/4A inhibitors, which fail predominantly due to the RAV R155K, the NS5A inhibitor daclatasvir has been observed to fail due to the growth of different RAVs in different individuals [37]. The RAVs Y93H/C/F/N have all been associated with daclatasvir resistance [9, 37]. Further, the RAVs are rarely detected pre-treatment but grow rapidly during treatment, indicating that they are present pre-treatment below detection limits [37]. To understand these observations, we applied our model to define the spectrum of mutants at the position 93 in the NS5A region of HCV.

**Fig 6:**
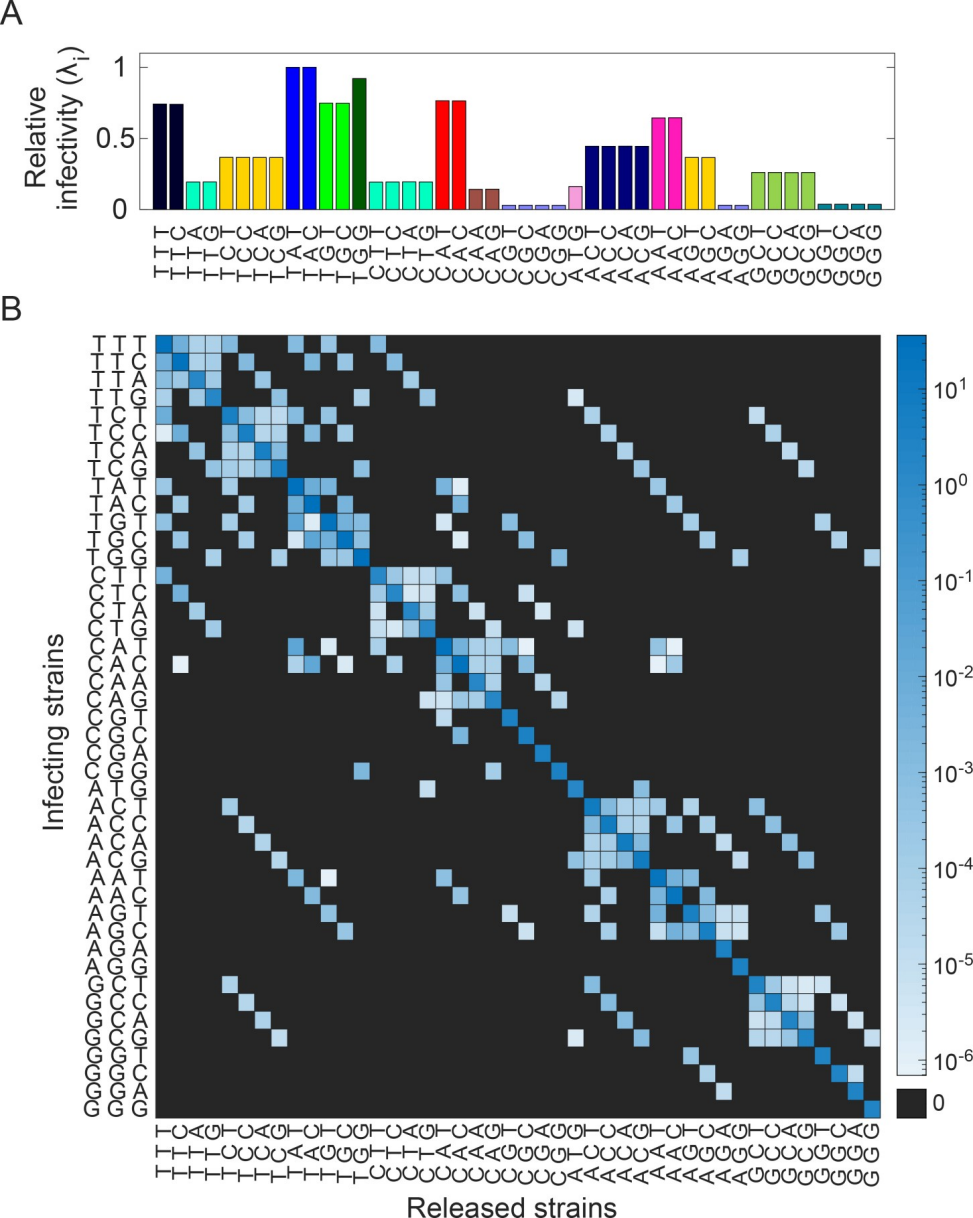
The relative infectivity and specific release rate of mutants at position 93 of the NS5A protein. **(A)** The probability of infection of virions carrying different codons, color coded by the amino acids they encode. **(B)** The specific release rate matrix summarized as a heat map from the data in S5 Fig.

**Fig 7:**
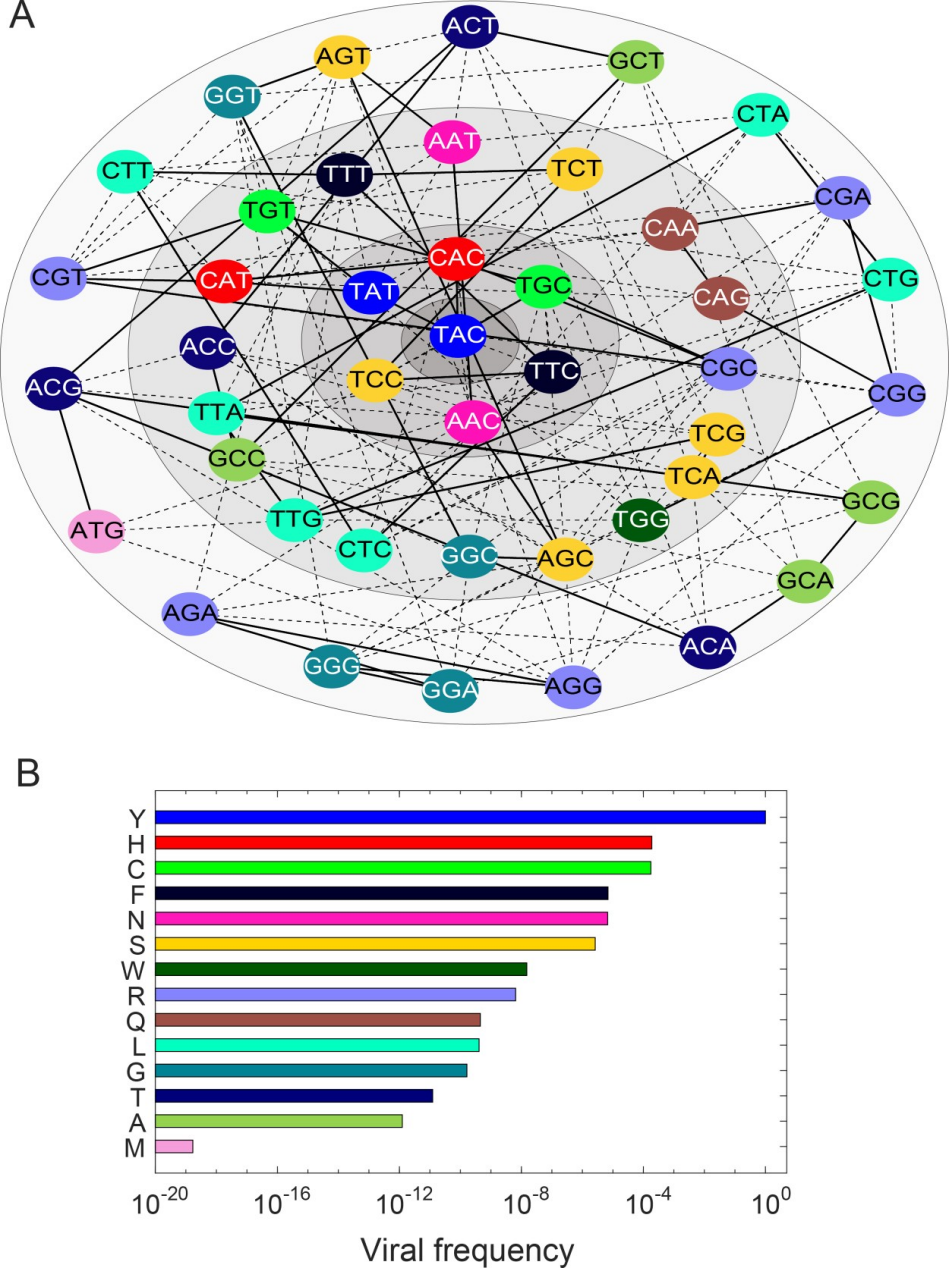
The mutant spectrum at position 93 of the NS5A protein. **(A)** The map of mutational pathways depicted circularly for compactness. Transitions are in solid lines and transversions in dashed lines. Annuli of increasingly lighter shades of grey represent increasing numbers of mutations from the founder strain, TAC, defining the amino acid Y at position 93 of the NS5A protein of HCV. **(B)** The frequencies of different variants at this position.

In a comprehensive study recently, the fitness of all single mutants, carrying every one of the 20 amino acids at every position of the NS5A protein, have been estimated experimentally [38]. We employed the fitness data pertaining to position 93 (S5 Fig). Based on amino acids with non-zero fitness, we found that 44 different codons could potentially exist at this locus in an infected individual. The problem is thus of a much larger scale than the R155 case above, where only 13 codons existed. To estimate the mutant frequencies, we first performed our stochastic intracellular simulations with each of the 44 codons as the infecting strain (S6 Fig) and estimated the relative infectivity (A) and the specific release rates (Fig 6B and S7 Fig). With these values, we solved our extracellular model and estimated the populations and frequencies of each of the variants at steady state. We constructed the mutational pathway map, involving 6 single mutants, 18 double mutants, and 19 triple mutants, connected via transitions and transversions (Fig 7A). The pathways explained the frequencies of the mutants we observed (Fig 7B).

Of the 6 single mutants, one was synonymous and thus contributed to the frequency of the wild-type amino acid, Y. Of the remaining 5 single mutants, 2, encoding the amino acids H and C, were produced by transitions of the wild-type codon, whereas the other three, encoding the amino acids F, N, and S, were produced by transversions. Indeed, H and C were the variants with the highest frequencies, ~0.01%. The next highest were F, N, and S, with frequencies of ~0.001%. The rest of the variants, involving 7 different amino acids, were present at lower frequencies ranging from 10^−8^–10^−12^. (One of the mutants, M, had a frequency far below 10^−12^, resulting in a mean virion number much less than 1 in a typical individual; the mutant is thus expected to occur rarely.) This distribution of frequencies thus defined the spectrum of mutants at the position 93 of NS5A within an HCV infected individual. It indicated that the Y93H and Y93C were most likely to be detected pre-treatment because of their high pre-treatment frequencies. The frequencies, however, were below detection limits of current assays, explaining why they are not typically detected. Similarly, RAVs containing each of the 13 amino acids are expected to exist in an infected individual below detection limits. The RAV that would lead to viral breakthrough during treatment would depend on the fitness of the RAVs in the presence of the drug, defined by the extent of resistance or increase in IC_50_ values relative to the wild-type [38]. The RAV with the most increase in IC_50_ may drive treatment failure. Thus, the wide spectrum of mutants renders a variety of resistance pathways accessible to the virus *in vivo*.

## Discussion

Treatment options for chronic hepatitis C are increasing rapidly as many new DAAs have been approved for clinical use recently and many are in advanced stages of development [11]. At the same time, the demand for DAAs is set to rise sharply with growing evidence of their success in the real world [39] and with >98% of the ~150 million chronic hepatitis C patients worldwide yet to receive DAA-based treatments [40, 41]. Efforts are therefore underway to develop rational strategies to identify the best combinations of the available DAAs, which would ensure cure while minimizing the treatment duration, cost, and side effects [2–7, 42, 43]. Our study informs these timely efforts. The success of DAAs relies on their ability to prevent the growth of resistance associated viral variants in patients [9]. In this study, we developed a multiscale mathematical model that quantifies the spectrum of such variants that may exist in chronically infected individuals, often below detection limits, before treatment initiation, and thus defines the possible pathways of the growth of drug resistance due to pre-existing variants. DAA combinations that most effectively preclude the realization of these pathways *in vivo* are likely to elicit the best responses.

Describing within-host HCV evolution has been an outstanding challenge, with many recent studies constructing multiscale models to integrate intracellular and extracellular dynamics [19, 20, 25, 31, 36, 44–46]. The complexity increases manifold because the evolution is strongly stochastic, given the mutation rate of approximately 10^−5^ per site per replication [25] and the small number of viral RNA, typically a few hundred, that an infected cell carries [27]. Stochastic models of HCV evolution have been constructed [25, 36]. The computational cost of such models increases prohibitively as the genome size or the viral and cell populations considered increases. Concepts such as the effective population size [47, 48] are then invoked to keep the simulations tractable, but this restricts the applicability of the models [48, 49]. Our study presents a novel strategy to overcome this limitation. We performed intracellular simulations fully stochastically and comprehensively, considering every possible genomic variant as the infecting strain. We thus obtained all possible expected “input-output relationships” for individual cells in an infected individual. These input-output relationships for all cells in the individual were coupled by the exchange of free virions through the plasma. Given that the population of free virions in a chronic hepatitis C patient is estimated to be over 10^10^ [18], the resulting extracellular dynamics could be solved deterministically. Our model thus gains accuracy over current models without a prohibitive escalation of computational cost.

The complexity in our model, resulting from the consideration of all possible genomic variants, is in keeping with recent advances in high throughput and single molecule experimentation. For the first time, a sizeable portion of the fitness landscape of HCV has recently been determined: In a *tour de force*, the fitness of every mutant of HCV in the NS5A region, obtained by replacing the amino acid at every residue in the protein with every one of the remaining 19 amino acids, one at a time, was estimated experimentally [38]. Further, advances in amplification, detection and sequencing technologies are allowing the identification of every genomic variant produced from an infected cell [50]. Our model is designed to efficiently exploit such data. Using a codon level description of amino acids, combinations of transitions and transversions that lead from any amino acid to each of the other 19 alternatives, a corresponding fitness landscape, and the input-output relationships above, we could predict the frequencies of all possible mutants at given loci, presenting a measure of the scale of the diversity of accessible mutational pathways. Thus, we estimated that 13 different amino acid variants encoded by 44 different codons would exist in the viral quasispecies in an infected individual at the residue 93 of the NS5A protein, presenting 44 different potential routes to NS5A inhibitor resistance. Our model estimated the frequencies of each of these variants and found them all to be below detection limits, highlighting the limitation of current assays and the importance of mathematical models in providing realistic estimates of RAV frequencies. Indeed, in a recent study using ultradeep sequencing, at the residue 31 of the NS5A protein, which is another locus of NS5A inhibitor resistance, although no resistance was detectable pre-treatment in one individual, 3 different RAVs, L31V, L31I and L31M, were detectable in the individual within a week of starting therapy that included the NS5A inhibitor daclatasvir [37].

We compared our estimate of the frequency of the RAV R155K, resistant to NS3/4A protease inhibitors, with corresponding database frequencies [33] and found good agreement, giving us further confidence in our formalism. We recognize that database frequencies are representative of sequences prevalent across patients and may be subject to selection pressures at the population level including transmission bottlenecks. Because we have considered databases collected before DAA treatments commenced, we expect transmitted drug resistance not to be a confounding factor. Further, transmission bottlenecks are expected to influence the viral envelope proteins much more strongly than nonstructural proteins. The database frequencies, which are estimated by sampling a large number of sequences across patients (here ~3000), are thus expected to broadly mimic the pre-treatment mutant frequencies at corresponding loci on nonstructural proteins in a typical patient. Future studies that may employ deeper sequencing techniques than currently available may provide a more direct test of our formalism.

Interestingly, we found that the rank ordering of the frequencies of the various mutants was not dictated by fitness effects alone, in contrast to the classical mutation-selection balance [21]. Strong founder effects offset the influence of fitness in our simulations. Combining the founder effects and the fitness landscape, we could create a map of mutational pathways accessible to any founder strain. Importantly, the maps were different for different founder strains containing the same amino acid but represented by different codons. Thus, HCV genotypes 1a and 1b both contain the amino acid R at the position 155 of the NS3 protein but have different mutational pathway maps because they are encoded by different codons. NS3/4A inhibitor resistance was thus predicted to be far more prevalent with genotype 1a than 1b, which is consistent with the rare detection of RAVs and the better response of the latter to NS3/4A inhibitor treatments [12, 51]. That the difference arises because genotype 1a requires a single transition whereas genotype 1b requires a transversion followed by a transition for the R155K mutation has been recognized earlier [36, 51]. Our model makes quantitative predictions of the frequencies of the mutant in the two cases, which is consistent with observations [33], facilitating more accurate tailoring of treatments for the two cases. Such tailoring may have to account also for the genetic backgrounds in which the RAVs arise, which may be different across the two genotypes, as has been recognized, for instance, with NS5A inhibitor resistance [8, 52, 53].

Currently, systematic resistance testing is not recommended before the start of DAA treatments, due possibly to the ability of DAAs to cure patients regardless of pre-existing RAVs [54]. Only RAVs with frequencies above ~10–15%, which are detectable using population sequencing techniques, have been found to influence treatment outcomes [55]. Our interest in estimating minority RAV frequencies is in optimizing treatments without compromising outcomes. We expect that dosages and/or treatment durations may be reduced beyond current guidelines if RAVs can be ensured to remain responsive with the altered protocols. Indeed, current guidelines do recommend resistance testing, where such testing is reliable and accessible, before the use of NS5A inhibitors [54]. Interestingly, a comprehensive analysis extending over 50 clinical trials showed recently that DAA treatments elicited better responses in treatment naïve individuals than in previous null responders to the combination of interferon and ribavirin [20]. A model based on the premise that greater responsiveness to interferon suppressed the replication space available to HCV and therefore prevented the growth of RAVs was able to quantitatively describe the clinical observations [20], reiterating the importance of RAVs in treatment optimization. By accurately estimating RAV frequencies, our model aids such optimization.

Many recent studies have detected RAVs in a significant fraction of patients pre-treatment [55–57]. This is not in conflict with our predictions of minority RAVs typically lying below detection limits. Where the fitness penalties associated with specific RAVs are not significant, it is possible that they exist well above detection limits. Thus, for instance, while RAVs were detected at the position Q30, no RAVs were detectable at the positions Y93 or L31, all associated with NS5A inhibitor resistance, in 41 HCV genotype 1a infected individuals or 77 HCV genotype 1a infected individuals coinfected with HIV [57]. RAV frequencies may increase in treatment experienced patients, given the weaker interferon responses expected in such individuals [20]. Further, transmitted resistance may also contribute to the observed pre-existence, especially with RAVs to NS5A inhibitors, which are known to last years in patients even in the absence of treatment [55].

We recognize that the identification of optimal DAA combinations requires additional inputs. In particular, the dynamics of the growth of RAVs during treatment must be accounted for. Remarkably, the extent of resistance, in terms of the fold change in IC_50_ relative to the wild-type, for every single amino acid variant in the NS5A region has been experimentally identified [38]. Extending our model by incorporating the latter data would present an understanding of the most likely pathways of the growth of pre-existing RAVs. A combination of high pre-existing frequency and high level of resistance would decide the most likely pathways. Drug combinations would then be designed to prevent those pathways. Such extensions of our model would also require knowledge of epistatic effects that define the fitness of viral genomes with multiple mutations, which is currently lacking for HCV. Techniques from statistical physics are being applied to develop more comprehensive fitness landscapes [58]. Further, resistance may often arise from new mutations that occur during treatment and not from the growth of pre-existing strains, in which case, either fully stochastic models [25] or models that estimate the waiting times for the emergence of such mutants [59] may have to be developed.

The dynamics during and post-treatment can be complex. In a recent study, the rate of viral load decline during treatment with a second-generation protease inhibitor, MK-5172, and the turnover of drug resistant variants post-treatment were found to be far more rapid than previously expected [16]. The study attributed the rapid decline to the cure of infected cells by the DAA. The rapid turnover of mutants post-treatment was argued to be due to cellular superinfection and the ensuing replacement of less fit strains by more fit ones within superinfected cells. This allowed a new, more fit strain to become dominant swiftly even when a less fit strain had established infection with maximal viremia leaving little “replication space” for the new mutant. HCV is thought to induce a superinfection block [60, 61], which renders such superinfection rare, although strains that exhibit enhanced ability to superinfect can be selected in vitro [35]. The mechanism of the replacement of the less fit strain by a more fit strain is less well understood [35]. Previous studies have speculated that the replacement may occur during cell division, when new replication space is created, and the more fit strain has an advantage in terms of establishing infection in the daughter cells [35, 62]. Models considering the partitioning of viral variants into daughter cells are yet to be constructed. Other immune mechanisms may also influence the dynamics during and post-treatment. For instance, the reduction in viral load due to treatment may reverse immune exhaustion and rejuvenate CD8+ T cell responses [43, 63–65]. This has been argued to contribute to the post-treatment cure of HCV infection in some patients despite detectable viremia at the end of treatment [43]. Whether this leads to responses against temporally dominant viral variants and contributes to the observed rapid turnover of variants remains to be examined. Further, cells that are cured by the treatment are likely to be exposed to interferon secreted when they were infected [66, 67]. Cells exposed to interferon may enter an antiviral state that renders their productive infection less likely [66–68]. HCV subverts this interferon response by introducing a block in translation [68]; the block is released when HCV is cleared and the cell cured [31, 68]. It is conceivable that fitter viral strains are more likely to overcome the interferon response in such cells and reestablish infection [31], which again may contribute to the rapid turnover of viral variants observed. Our study has focused on the frequencies of mutants before the onset of treatment, which are less likely to be influenced by these latter mechanisms.

We envision broader implications of our study. The prevalent paradigm for describing within-host viral evolution is the molecular quasispecies theory [69, 70]. The theory, built originally to describe the origin of life, has shaped the modern view of viral evolution by describing the error-prone self-replication of molecules such as RNA, which constitute viral genomes. The theory, however, assumes a well-mixed milieu of such genomes subjected to common selection forces, which ceases to hold for viruses such as HCV where intracellular and extracellular selection are segregated. Our model thus goes beyond models based on the molecular quasispecies theory [19, 20, 45] by accurately describing and integrating intracellular and extracellular evolution. The resulting formalism may be useful in describing the within-host evolution of other important human viruses, such as dengue, West Nile and Zika, which have a lifecycle similar to HCV. A second implication of our formalism is in vaccine design. Although we have focused here on loci leading to drug resistance, our model can be readily applied to sites of immune escape, allowing estimation of the genetic diversity that vaccine candidates must target [58].

In summary, our study presents a novel approach to estimating the entire spectrum of mutants present in infected individuals, explains several clinical observations associated with chronic hepatitis C, and presents a framework that would aid the rational optimization of modern DAA-based treatments.

## Methods

### Mathematical model

We present details here of our multiscale model of within-host HCV dynamics and evolution (Fig 1).

#### Intracellular dynamics

A schematic of the intracellular model is in Fig 1C. We first considered a cell infected with a virion carrying a genome of type *j*, where *j* was either the wild-type, denoted *j =* 0, or one of the 4^*L*^-1 mutants, when *L* sites constituted the resistance locus. When we considered the 3 positions of a codon at a particular residue, *i*.*e*., *L* = 3, it followed that *j*∈{0,1,2,…,63}. The genome was assumed to be released into the cytoplasm, where it could replicate to a negative strand genome of type *i*∈{0,1,2,…,63}, yielding a replication complex, which in turn could act as a template for producing more positive strand genomes from among the 64 possible variants. Specifically, positive strand genomes *j* replicated at the per capita rate *k*_+_*f*_*j*_ constrained by a logistic term that restricted the maximum number of positive and negative strand genomes to the carrying capacity *K*. *f*_*j*_ was the fitness of genome *j* relative to the wild-type. One such replication event yielded the genome *i* with the probability $H_{ij}=\mu_{ts}{}^{N_{ts}}\mu_{tv}{}^{N_{tv}}{\left(1-\mu_{ts}-2\mu_{tv}\right)}^{L-N_{ts}-N_{tv}}$, where of the *L* sites, genomes *i* and *j* differed by *N*_*ts*_ transitions and *N*_*tv*_ transversions, which occurred with probabilities *μ*_*ts*_ and *μ*_*tv*_ per site, respectively. A replication complex *i* in turn replicated at the rate *k*_*f*_*i*_ and yielded a positive strand genome *k* with the probability *H*_*ki*_. The genomes and the replication complexes could degrade at the per capita rates *d*_*RNA*_ and *d*_*RC*_, respectively. The positive strand genomes could also be packaged and released as progeny virions at the per capita rate *ρ*. These events and their rates are summarized in Table 2.

**Table 2:** Events in the intracellular model along with their rates. The symbols and their meanings are described in the text. Parameter values are in Table 1.

Description	Event	Rate
Replication of RNA to RC	*RNA*_*j*_→*RC*_*i*_	$k_{+}\left(1-\frac{T_{RNA}+T_{RC}}{K}\right)H_{ij}f_{j}RNA_{j}$
RNA degradation	*RNA*_*j*_→*ϕ*	*d*_*RNA*_*RNA*_*j*_
RNA assembly and release	*RNA*_*j*_→*V*_*j*_	*ρRNA*_*j*_
Replication of RC to RNA	*RC*_*j*_→*RNA*_*i*_+*RC*_*j*_	$k_{-}\left(1-\frac{T_{RNA}+T_{RC}}{K}\right)H_{ij}f_{j}RC_{j}$
RC degradation	*RC*_*j*_→*ϕ*	*d*_*RC*_*RC*_*j*_

Using a set of parameter values representative of HCV infection (Methods, Table 1), we simulated these events stochastically using the Gillespie algorithm [71]. We performed simulations for a duration τ = 72 h, representing the mean lifetime of an infected cell, based on the range (0.014–0.5 d^-1^) of infected cell loss rates estimated previously [28]. (The mean infected cell loss rates have been found to vary across studies [72–74]. Using longer infected cell lifespans, accordingly, did not significantly alter our findings (S8 Fig).) For any genome *j* as the infecting strain, we repeated our simulations 10^6^ times. We computed the probability *λ*_*j*_ with which the strain *j* would result in productive infection as the fraction of realizations in which at least one progeny virion was released relative the corresponding fraction for the wild-type (or the fittest genome). When productive infection occurred, we also obtained the distribution of progeny virions released, *p*_*ij*_, which we termed the specific release rate, as the number of virions of type *i* released on average from cells infected by genome *j* divided by the lifetime *τ*. We repeated the simulations for each of the 64 genomes as the infecting strain and estimated *λ*_*j*_ and *p*_*ij*_. We employed these quantities to describe the extracellular viral kinetics.

#### Extracellular dynamics

Modifying the architecture of the basic model of viral kinetics [17] using the quantities above, we constructed the following equations to describe the extracellular viral kinetics (Fig 1D):

$$\begin{array}{c} \frac{dT}{dt}=s_{gen}+k_{prt}T\left(1-\frac{T+\sum_{i}I_{i}+N}{K_{cell}}\right)-d_{T}T-\sum_{i}\beta \lambda_{i}V_{i}T\\ \frac{dI_{i}}{dt}=\beta \lambda_{i}V_{i}T+k_{pri}I_{i}\left(1-\frac{T+\sum_{i}I_{i}+N}{K_{cell}}\right)-\delta I_{i}\\ \frac{dV_{i}}{dt}=\sum_{j}p_{ij}I_{j}-cV_{i} \end{array}$$ (1)

Here, uninfected hepatocytes that are targets of infection, *T*, are produced at the rate *s*_*gen*_, are lost at the per capita rate *d*_*T*_, and proliferate at the per capita rate *k*_*prt*_, the latter restricted by the logistic term that limits the total hepatocyte population to *K*_*cell*_. *N* represents cells that are not targets of infection, due, for instance, to inadequate expression of entry receptors [75]. The target cells, *T*, are infected by virions *V*_*i*_, carrying genomes *i*, at the per capita rate *βλ*_*i*_*V*_*i*_, where *λ*_*i*_ is the probability with which genome *i* infects a cell relative to the wild-type (or the fittest genome), identified by the simulations above, and *β* is the second order rate constant of the infection of target cells with wild-type virions. Summation over *i* thus yielded the total rate of loss of target cells due to infection.

We defined *I*_*i*_ as the population of cells productively infected with virions *V*_*i*_. These cells proliferated at the per capita rate *k*_*pri*_ restricted by the logistic term above and were lost at the per capita rate δ. Cells *I*_*j*_ produced virions *V*_*i*_ at the per cell rate *p*_*ij*_, the specific release rate identified from the intracellular simulations above. Free virions are cleared at the per capita rate *c*.

Solving the above equations for steady state yielded the frequencies of all variants, quantifying the spectrum of mutants.

### Parameter estimates and solution of model equations

We obtained most parameter values from previous studies (Table 1). We estimated the replication rates, *k*_+_ and *k*_-_, and the carrying capacity, *K*, to ensure consistency with the overall population dynamics of viral RNA in cells (S1 Text, S9 Fig). We performed simulations of intracellular dynamics using the Stochastic Simulation Algorithm (SSA) in the software Stochkit 2 [76]. We ensured that 10^6^ simulations were adequate to obtain reliable predictions (S10 Fig). We solved our equations of extracellular dynamics in MATLAB using initial conditions where the target cells were in their uninfected steady state, infected cells were absent and a single virion of the wild-type existed.

## Supporting information

S1 Fig Mutant spectrum at position 155 of NS3 using in vitro fitness estimates. Frequencies of the different mutants at steady state estimated as in Fig 4 but using the fitness of different RAVs estimated in vitro (inset) [34]. Shown for comparison is the database value of the mutant R155K for HCV genotype 1a (red dot) [33].
(PDF)

S2 Fig Founder effects in the two-locus/two-allele model. We performed stochastic simulations of intracellular evolution using a two-locus/two-allele model. We thus had 4 genomes: the wild-type, two single mutants, and the double mutant. For simplicity, we let the single mutants have the same relative fitness, *f* = 0.9, and let the double mutant have the fitness, *f*^2^, representing a multiplicative fitness landscape. Using each of these strains as the infecting strain, we ran simulations for *τ* = 72 h and estimated the populations of different genomes and replication complexes as well as the virions released. The populations of wild-type (blue), single mutant (red), and double mutant (green) strains are shown when the infecting strain is the **(A-C)** wild-type, **(D-F)** single mutant, and **(G-I)** double mutant. Solid lines are means and dashed lines standard deviations. Consistent with our calculations in Fig 2, the infecting strain dominated the populations and strains removed by more than one mutation from the infecting strain were hardly produced.
(PDF)

S3 Fig Specific release rate for NS3 position 155. The average rate at which virions carrying different codons are released following infection of a cell with a virion carrying the codon mentioned in the panels. The parameters are those used in Fig 3.
(PDF)

S4 FigComparison with previous models.Previous models (e.g., [18]) (light bars) underpredict mutant frequencies in comparison with the present model (dark bars). We estimated the mutant frequencies from previous models for **(A)** NS3 position 155 and **(B)** NS5A position 93 using the following equations: $\frac{dT}{dt}=s_{gen}+k_{prt}T\left(1-\frac{T+\sum_{i}I_{i}+N}{K_{cell}}\right)-d_{T}T-\sum_{i}\beta V_{i}T$; $\frac{dI_{i}}{dt}=\beta V_{i}T+k_{pri}I_{i}\left(1-\frac{T+\sum_{i}I_{i}+N}{K_{cell}}\right)-\delta I_{i}$; and $\frac{dV_{i}}{dt}=p\sum_{j}H_{ij}f_{j}I_{j}-cV_{i}$, where the terms have the same meanings as those in Eq (1) of the main text. The fitness *f*_*j*_ and the mutation probability *H*_*ij*_ are identical to those used in our model (see Methods). Each infected cell is assumed to produce genomes in proportion to the fitness of the infecting strain. Mutants are produced from the cell in proportion to the probability that the infecting strain yields the respective mutants during one round of replication. The previous models thus do not account for stochastic intracellular evolution and the associated founder effects, which leads to the underprediction of mutants.(PDF)

S5 Fig Relative fitness of genomes at amino acid position 93 in the NS5A region of HCV. The fitness values have been extracted from the data reported recently [38].
(PDF)

S6 Fig Intracellular dynamics and evolution leading to NS5A resistance. The averaged evolution of the populations of genomes carrying different codons following infection with TAC at the position 93 of the NS5A region of HCV.
(PDF)

S7 Fig Specific release rate for NS5A position 93. The average rate at which virions carrying different codons are released following infection of a cell with a virion carrying the codon mentioned in the panels. The parameters are those used in Fig 6. The figure extends over 3 pages.
(PDF)

S8 Fig Influence of infected cell lifespan. We extended the simulations in Fig 2D to longer durations corresponding to the lower infected cell death rates estimated in some studies (0.14 d^-1^ [72]). The mean viruses released do not change significantly from that at 72 h used in Fig 2D, the two lifespans indicated using dashed lines.
(PDF)

S9 Fig Estimation of unknown parameters. Stochastic simulations based on the model of S1 Text showing the overall RNA (blue) and RC (red) populations in infected cells with *K* = 270 (solid lines) and *K* = 300 (dashed lines). The horizontal dotted lines mark the steady state values of 200 and 40 for RNA and RCs, respectively. With *K* = 270, the steady state values are reached by 48 h (vertical dotted line), as observed experimentally, but not so with other values of *K*.
(PDF)

S10 Fig Effect of the number of realizations. The time-evolution of wild-type (green), single mutant (red) and double mutant (blue) following infection with the single mutant strain obtained by averaging 10^6^ (left) and 10^5^ (right) realizations in our two-locus/two-allele model (S2 Fig). Averages are reliably obtained with 10^6^ realizations. The other parameters are the same as in S2 Fig.
(PDF)

S1 Table Specific release rate matrix. (PDF)

S1 Text Estimation of unknown parameters. (PDF)
