# Supplementary material for: Mutational pathway maps and founder effects define the within-host spectrum of hepatitis C virus mutants resistant to drugs
Source: PLoS Pathog. 2019 Apr 1;15(4):e1007701. doi: 10.1371/journal.ppat.1007701 (PMC6459561; doi:10.1371/journal.ppat.1007701)
Supplement: S1 Fig — Frequencies of the different mutants at steady state estimated as in Fig 4 but using the fitness of different RAVs estimated in vitro (inset) [34]. Shown for comparison is the database value of the mutant R155K for HCV genotype 1a (red dot) [33]. (PDF) [file ppat.1007701.s001.pdf]

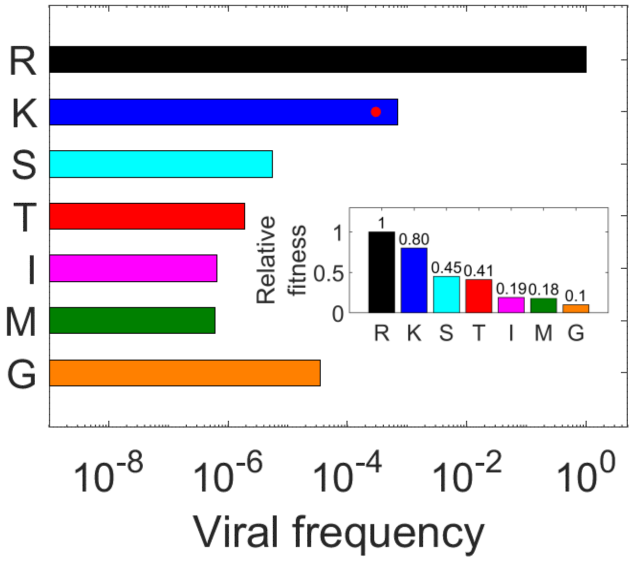

S1 Figure. Mutant spectrum at position 155 of NS3 using in vitro fitness estimates. Frequencies of the different mutants at steady state estimated as in Fig. 4 but using the fitness of different RAVs estimated in vitro (inset) [34]. Shown for comparison is the database value of the mutant R155K for HCV genotype 1a (red dot) [33].
