# Supplementary material for: Mutational pathway maps and founder effects define the within-host spectrum of hepatitis C virus mutants resistant to drugs
Source: PLoS Pathog. 2019 Apr 1;15(4):e1007701. doi: 10.1371/journal.ppat.1007701 (PMC6459561; doi:10.1371/journal.ppat.1007701)
Supplement: S2 Fig — We performed stochastic simulations of intracellular evolution using a two-locus/two-allele model. We thus had 4 genomes: the wild-type, two single mutants, and the double mutant. For simplicity, we let the single mutants have the same relative fitness, f = 0.9, and let the double mutant have the fitness, f2, representing a multiplicative fitness landscape. Using each of these strains as the infecting strain, we ran simulations for τ = 72 h and estimated the populations of different genomes and replication complexes as well as the virions released. The populations of wild-type (blue), single mutant (red), and double mutant (green) strains are shown when the infecting strain is the (A-C) wild-type, (D-F) single mutant, and (G-I) double mutant. Solid lines are means and dashed lines standard deviations. Consistent with our calculations in Fig 2, the infecting strain dominated the populations and strains removed by more than one mutation from the infecting strain were hardly produced. (PDF) [file ppat.1007701.s002.pdf]

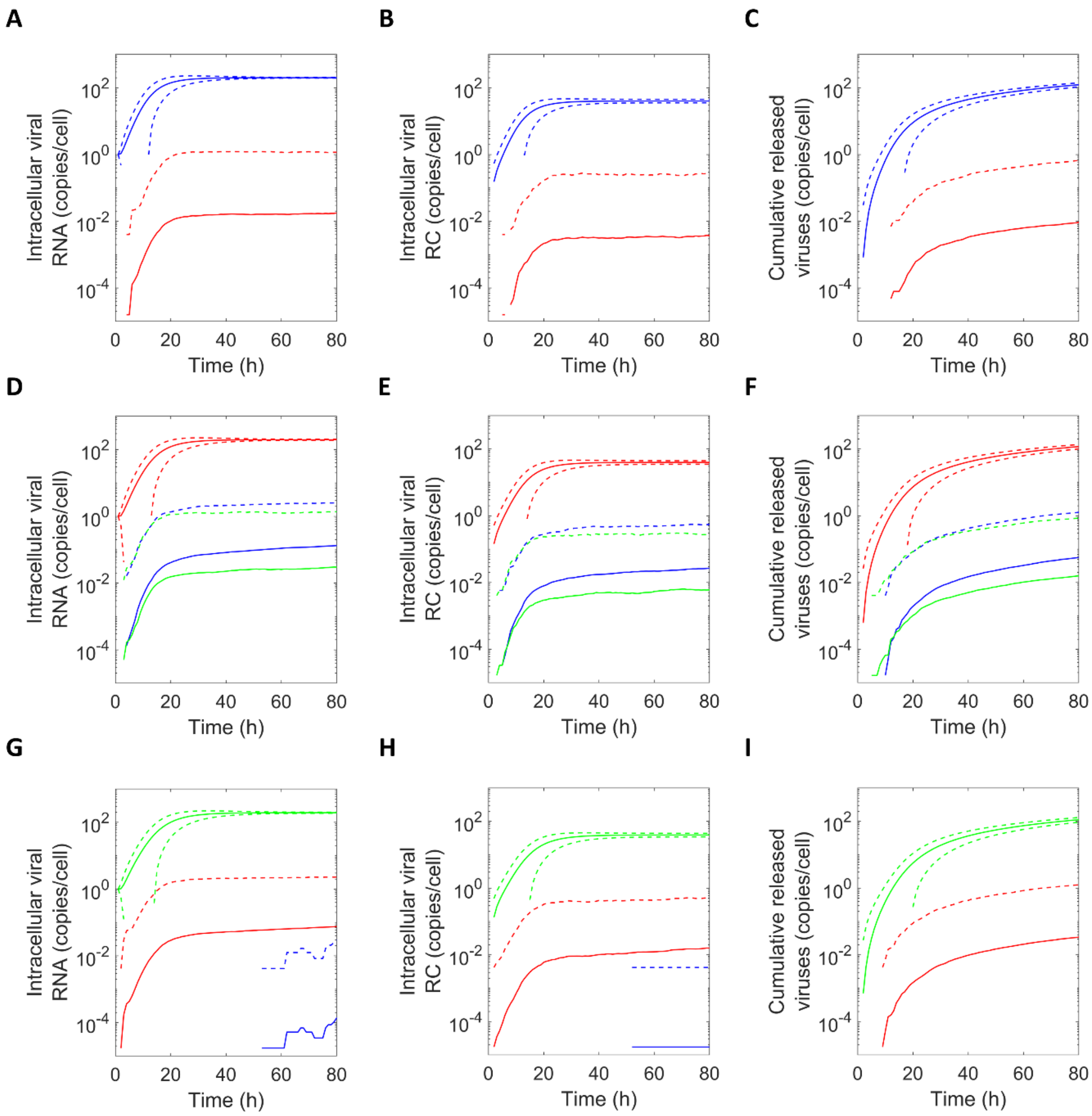

S2 Figure. Founder effects in the two-locus/two-allele model. We performed stochastic simulations of intracellular evolution using a two-locus/two-allele model. We thus had 4 genomes: the wild-type, two single mutants, and the double mutant. For simplicity, we let the single mutants have the same relative fitness, f=0.9, and let the double mutant have the fitness, f^2, representing a multiplicative fitness landscape. Using each of these strains as the infecting strain, we ran simulations for τ=72 h and estimated the populations of different genomes and replication complexes as well as the virions released. The populations of wild-type (blue), single mutant (red), and double mutant (green) strains are shown when the infecting strain is the **(A-C)** wild-type, **(D-F)** single mutant, and **(G-I)** double mutant. Solid lines are means and dashed lines standard deviations. Consistent with our calculations in Fig. 2, the infecting strain dominated the populations and strains removed by more than one mutation from the infecting strain were hardly produced.
