# Supplementary material for: Mutational pathway maps and founder effects define the within-host spectrum of hepatitis C virus mutants resistant to drugs
Source: PLoS Pathog. 2019 Apr 1;15(4):e1007701. doi: 10.1371/journal.ppat.1007701 (PMC6459561; doi:10.1371/journal.ppat.1007701)
Supplement: S3 Fig — The average rate at which virions carrying different codons are released following infection of a cell with a virion carrying the codon mentioned in the panels. The parameters are those used in Fig 3. (PDF) [file ppat.1007701.s003.pdf]

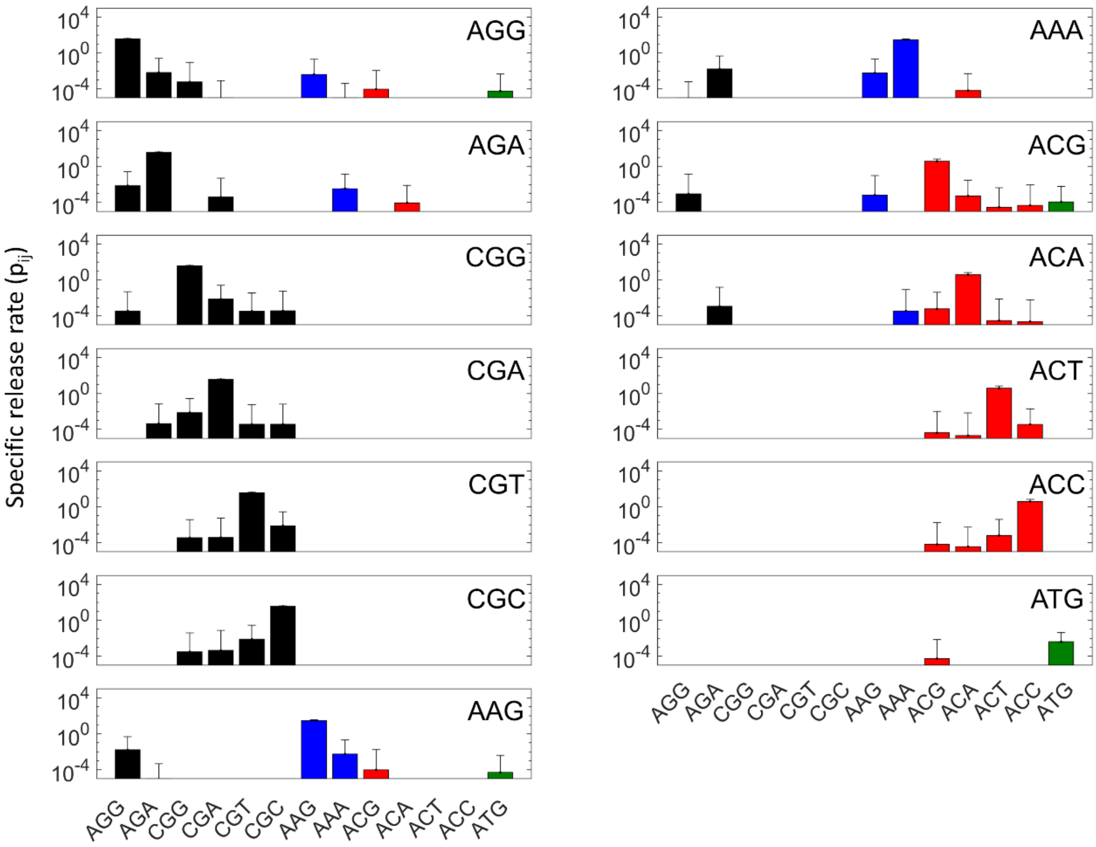

S3 Figure. Specific release rate for NS3 position 155. The average rate at which virions carrying different codons are released following infection of a cell with a virion carrying the codon mentioned in the panels. The parameters are those used in Fig. 3.
