# Supplementary material for: Mutational pathway maps and founder effects define the within-host spectrum of hepatitis C virus mutants resistant to drugs
Source: PLoS Pathog. 2019 Apr 1;15(4):e1007701. doi: 10.1371/journal.ppat.1007701 (PMC6459561; doi:10.1371/journal.ppat.1007701)
Supplement: S4 Fig — Previous models (e.g., [18]) (light bars) underpredict mutant frequencies in comparison with the present model (dark bars). We estimated the mutant frequencies from previous models for (A) NS3 position 155 and (B) NS5A position 93 using the following equations: dTdt=sgen+kprtT(1−T+∑iIi+NKcell)−dTT−∑iβViT; dIidt=βViT+kpriIi(1−T+∑iIi+NKcell)−δIi; and dVidt=p∑jHijfjIj−cVi, where the terms have the same meanings as those in Eq (1) of the main text. The fitness fj and the mutation probability Hij are identical to those used in our model (see Methods). Each infected cell is assumed to produce genomes in proportion to the fitness of the infecting strain. Mutants are produced from the cell in proportion to the probability that the infecting strain yields the respective mutants during one round of replication. The previous models thus do not account for stochastic intracellular evolution and the associated founder effects, which leads to the underprediction of mutants. (PDF) [file ppat.1007701.s004.pdf]

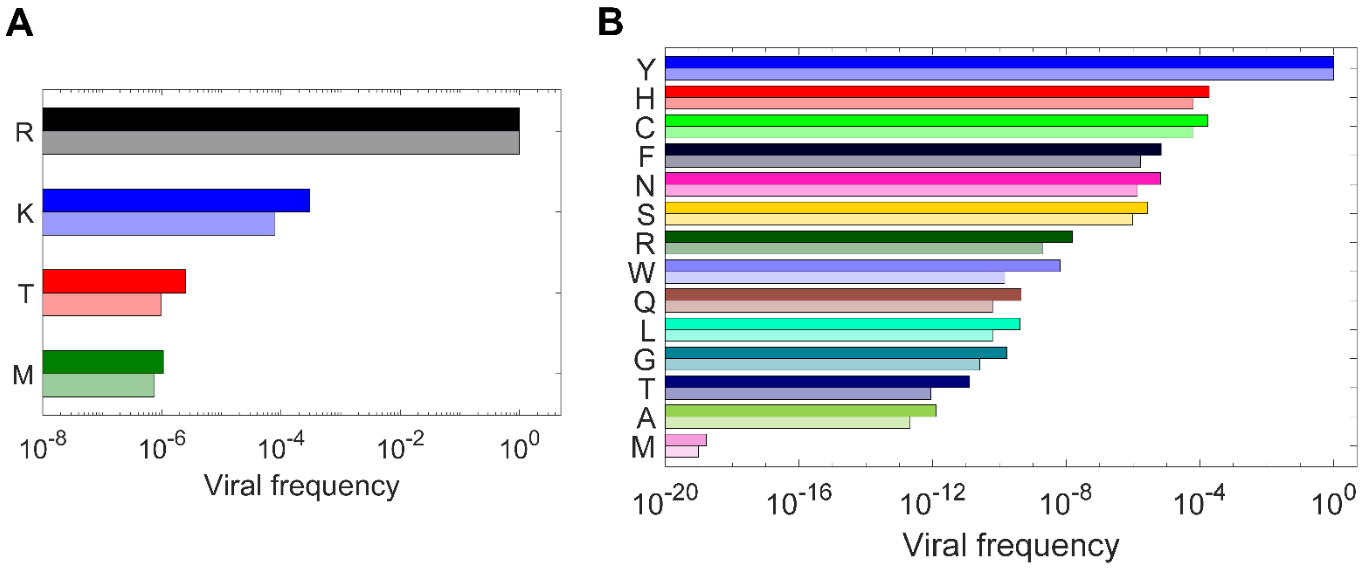


**S4 Figure. Comparison with previous models.** Previous models (e.g., [18]) (light bars) underpredict mutant frequencies in comparison with the present model (dark bars). We estimated the mutant frequencies from previous models for **(A)** NS3 position 155 and **(B)** NS5A position 93 using the following equations:

$$\frac{dT}{dt} = s_{gen} + k_{prt}T\left(1 - \frac{T + \sum_i I_i + N}{K_{cell}}\right) - d_T T - \sum_i \beta V_i T \;;$$

$$\frac{dI_i}{dt} = \beta V_i T + k_{pri} I_i \left(1 - \frac{T + \sum_i I_i + N}{K_{cell}}\right) - \delta I_i \;;$$ and $$\frac{dV_i}{dt} = p\sum_j H_{ij} f_j I_j - cV_i \,,$$ where the terms have the same meanings as those in Eq. (1) of the main text. The fitness $f_j$ and the mutation probability $H_{ij}$ are identical to those used in our model (see Methods). Each infected cell is assumed to produce genomes in proportion to the fitness of the infecting strain. Mutants are produced from the cell in proportion to the probability that the infecting strain yields the respective mutants during one round of replication. The previous models thus do not account for stochastic intracellular evolution and the associated founder effects, which leads to the underprediction of mutants.
