# Supplementary material for: Mutational pathway maps and founder effects define the within-host spectrum of hepatitis C virus mutants resistant to drugs
Source: PLoS Pathog. 2019 Apr 1;15(4):e1007701. doi: 10.1371/journal.ppat.1007701 (PMC6459561; doi:10.1371/journal.ppat.1007701)
Supplement: S5 Fig — The fitness values have been extracted from the data reported recently [38]. (PDF) [file ppat.1007701.s005.pdf]

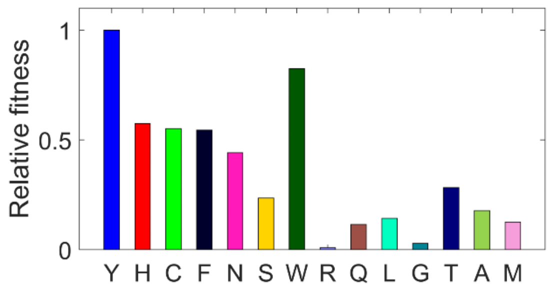

S5 Figure. Relative fitness of genomes at amino acid position 93 in the NS5A region of HCV. The fitness values have been extracted from the data reported recently [38].
