# Supplementary material for: Mutational pathway maps and founder effects define the within-host spectrum of hepatitis C virus mutants resistant to drugs
Source: PLoS Pathog. 2019 Apr 1;15(4):e1007701. doi: 10.1371/journal.ppat.1007701 (PMC6459561; doi:10.1371/journal.ppat.1007701)
Supplement: S6 Fig — The averaged evolution of the populations of genomes carrying different codons following infection with TAC at the position 93 of the NS5A region of HCV. (PDF) [file ppat.1007701.s006.pdf]

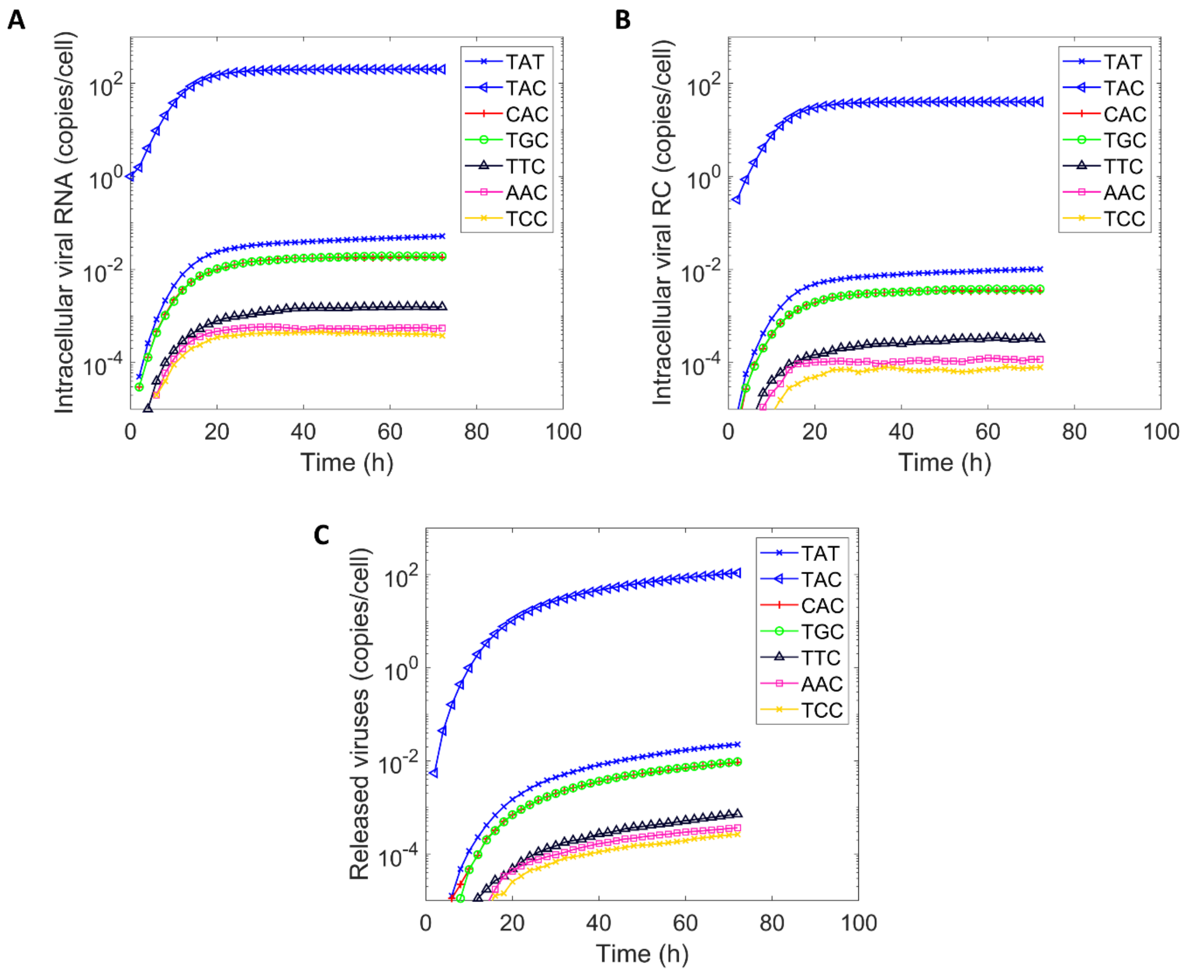

S6 Figure. Intracellular dynamics and evolution leading to NS5A resistance. The averaged evolution of the populations of genomes carrying different codons following infection with TAC at the position 93 of the NS5A region of HCV.
