# Supplementary material for: Mutational pathway maps and founder effects define the within-host spectrum of hepatitis C virus mutants resistant to drugs
Source: PLoS Pathog. 2019 Apr 1;15(4):e1007701. doi: 10.1371/journal.ppat.1007701 (PMC6459561; doi:10.1371/journal.ppat.1007701)
Supplement: S7 Fig — The average rate at which virions carrying different codons are released following infection of a cell with a virion carrying the codon mentioned in the panels. The parameters are those used in Fig 6. The figure extends over 3 pages. (PDF) [file ppat.1007701.s007.pdf]

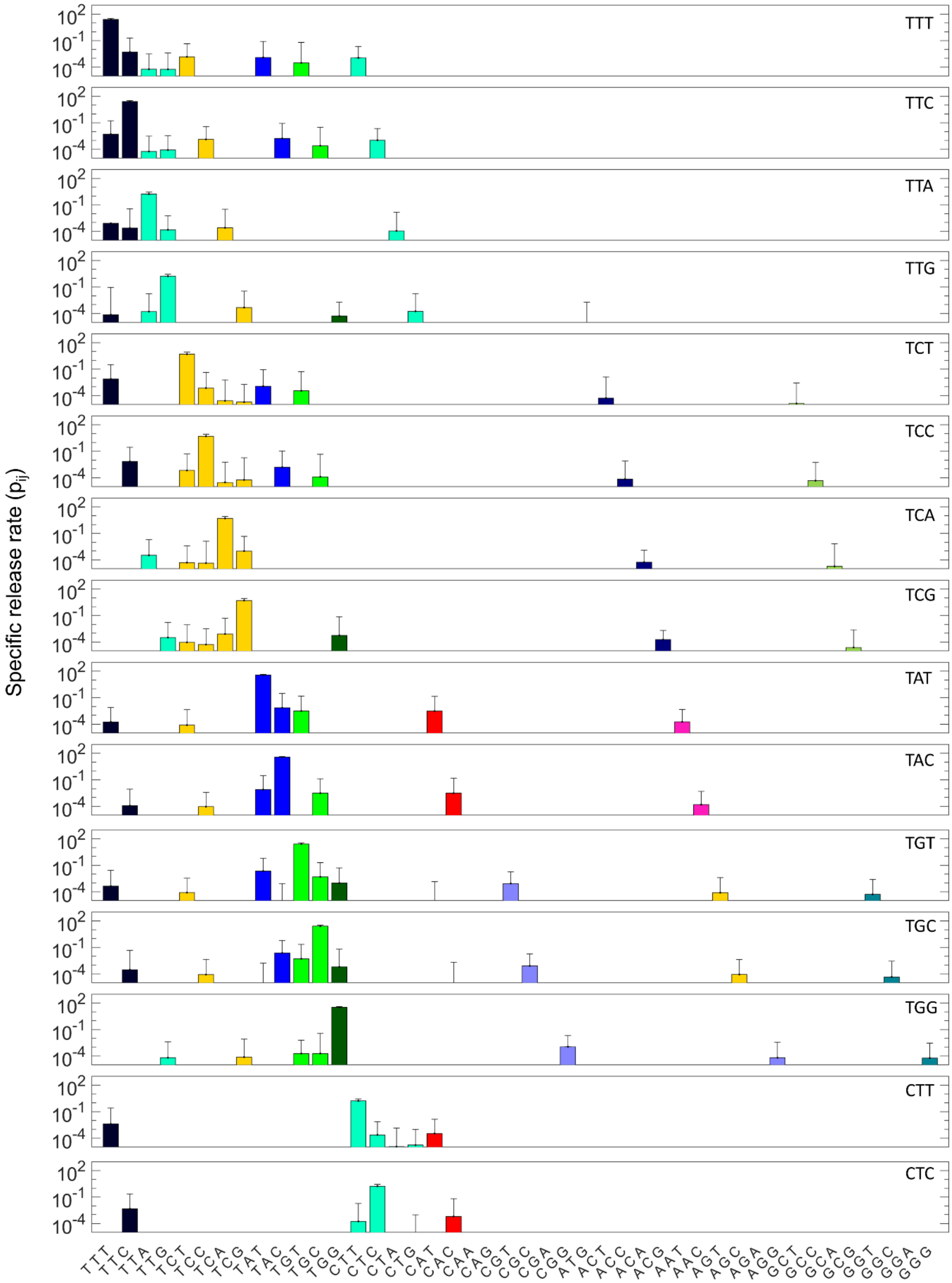
Specific release rate (p_{ij})
TTT
TTC
TTA
TTG
TCT
TCC
TCA
TCG
TAT
TAC
TGT
TGC
TGG
CTT
CTC
10^2
10^{-1}
10^{-4}
TTT TTC TTA TTG TCT TCC TCA TCG TAT TAC TGT TGC TGG CTT CTC CTA CTG CAT CAC CAA CAG CGT CGC CGA CGG ATG ACT ACC ACA ACG AAT AAC AGT AGC AGA AGG GCT GCC GCA GCG GGT GGC GGA GGG

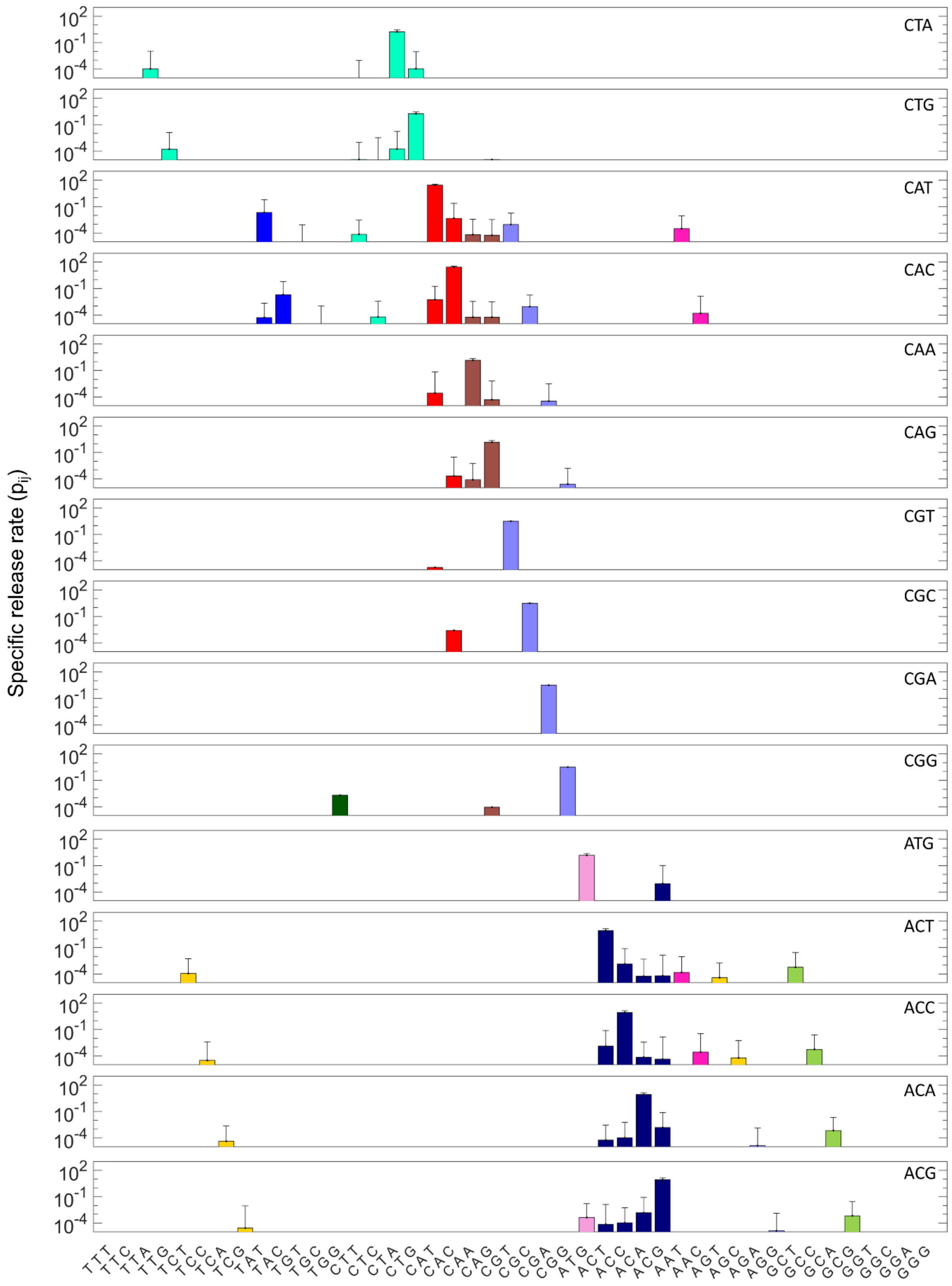
Specific release rate (p_{ij})
CTA
CTG
CAT
CAC
CAA
CAG
CGT
CGC
CGA
CGG
ATG
ACT
ACC
ACA
ACG
10^2
10^{-1}
10^{-4}
TTT TTC TTA TTG TCT TCC TCA TCG TAT TAC TGT TGC TGG CTT CTC CTA CTG CAT CAC CAA CAG CGT CGC CGA CGG ATG ACT ACC ACA ACG AAT AAC AGT AGC AGA AGG GCT GCC GCA GCG GGT GGC GGA GGG

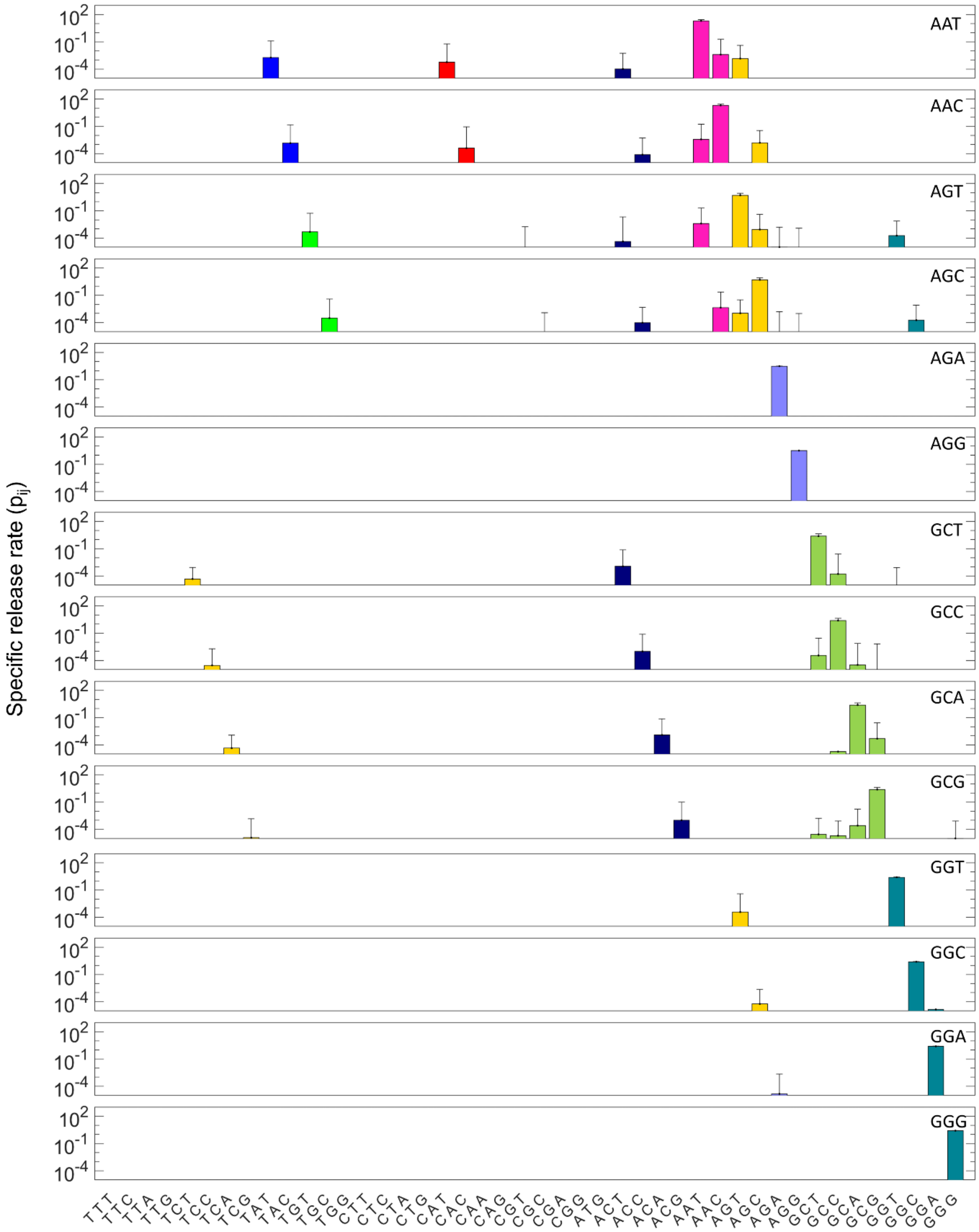

S7 Figure. Specific release rate for NS5A position 93. The average rate at which virions carrying different codons are released following infection of a cell with a virion carrying the codon mentioned in the panels. The parameters are those used in Fig. 6. The figure extends over 3 pages.
