# Supplementary material for: Mutational pathway maps and founder effects define the within-host spectrum of hepatitis C virus mutants resistant to drugs
Source: PLoS Pathog. 2019 Apr 1;15(4):e1007701. doi: 10.1371/journal.ppat.1007701 (PMC6459561; doi:10.1371/journal.ppat.1007701)
Supplement: S8 Fig — We extended the simulations in Fig 2D to longer durations corresponding to the lower infected cell death rates estimated in some studies (0.14 d-1 [72]). The mean viruses released do not change significantly from that at 72 h used in Fig 2D, the two lifespans indicated using dashed lines. (PDF) [file ppat.1007701.s008.pdf]

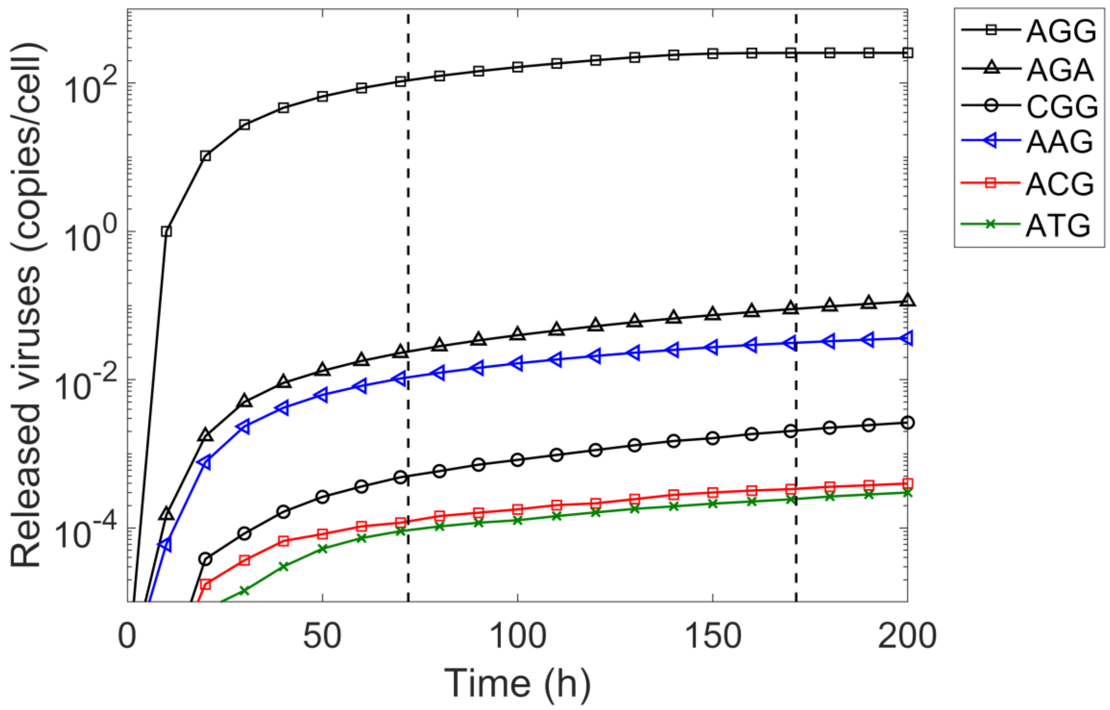

S8 Figure. Influence of infected cell lifespan. We extended the simulations in Fig. 2D to longer durations corresponding to the lower infected cell death rates estimated in some studies (0.14 d^{-1} [72]). The mean viruses released do not change significantly from that at 72 h used in Fig. 2D, the two lifespans indicated using dashed lines.
