# Supplementary material for: Mutational pathway maps and founder effects define the within-host spectrum of hepatitis C virus mutants resistant to drugs
Source: PLoS Pathog. 2019 Apr 1;15(4):e1007701. doi: 10.1371/journal.ppat.1007701 (PMC6459561; doi:10.1371/journal.ppat.1007701)
Supplement: S9 Fig — Stochastic simulations based on the model of S1 Text showing the overall RNA (blue) and RC (red) populations in infected cells with K = 270 (solid lines) and K = 300 (dashed lines). The horizontal dotted lines mark the steady state values of 200 and 40 for RNA and RCs, respectively. With K = 270, the steady state values are reached by 48 h (vertical dotted line), as observed experimentally, but not so with other values of K. (PDF) [file ppat.1007701.s009.pdf]

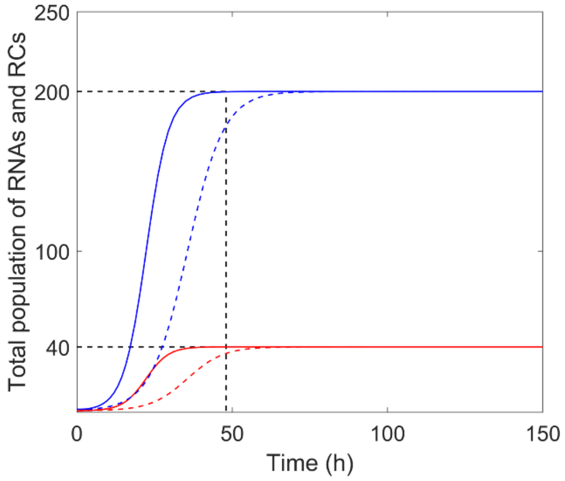

S9 Figure. Estimation of unknown parameters. Stochastic simulations based on the model of S1 Text showing the overall RNA (blue) and RC (red) populations in infected cells with *K*=270 (solid lines) and *K*=300 (dashed lines). The horizontal dotted lines mark the steady state values of 200 and 40 for RNA and RCs, respectively. With *K*=270, the steady state values are reached by 48 h (vertical dotted line), as observed experimentally, but not so with other values of *K*.
