# Supplementary material for: Mutational pathway maps and founder effects define the within-host spectrum of hepatitis C virus mutants resistant to drugs
Source: PLoS Pathog. 2019 Apr 1;15(4):e1007701. doi: 10.1371/journal.ppat.1007701 (PMC6459561; doi:10.1371/journal.ppat.1007701)
Supplement: S10 Fig — The time-evolution of wild-type (green), single mutant (red) and double mutant (blue) following infection with the single mutant strain obtained by averaging 106 (left) and 105 (right) realizations in our two-locus/two-allele model (S2 Fig). Averages are reliably obtained with 106 realizations. The other parameters are the same as in S2 Fig. (PDF) [file ppat.1007701.s010.pdf]

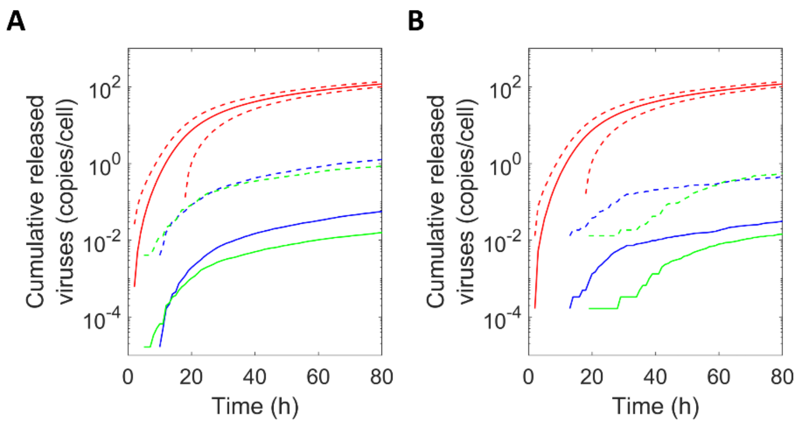


**S10 Figure. Effect of the number of realizations.** The time-evolution of wild-type (green), single mutant (red) and double mutant (blue) following infection with the single mutant strain obtained by averaging $10^6$ (left) and $10^5$ (right) realizations in our two-locus/two-allele model (S2 Fig.). Averages are reliably obtained with $10^6$ realizations. The other parameters are the same as in S2 Fig.
