# Supplementary material for: Mutational pathway maps and founder effects define the within-host spectrum of hepatitis C virus mutants resistant to drugs
Source: PLoS Pathog. 2019 Apr 1;15(4):e1007701. doi: 10.1371/journal.ppat.1007701 (PMC6459561; doi:10.1371/journal.ppat.1007701)
Supplement: S1 Table — (PDF) [file ppat.1007701.s011.pdf]

S1 Table. Specific release rate matrix. The mean ± standard deviation specific release rates shown as the heat map in Fig. 3C. The mutants are colour-coded base on the amino-acid type. The diagonal elements shown in light blue represent the release of the infecting strains. The other non-zero entries are in grey.

Released strain

Infecting strain	AGG	AGA	CGG	CGA	CGT	CGC	AAG	AAA	ACG	ACA	ACT	ACC	ATG
AGG	36 ± 6	0 ± 0.3	0 ± 0.05	0	0	0	0 ± 0.2	0	0 ± 0.003	0	0	0	0 ± 0.003
AGA	0 ± 0.2	36 ± 6	0	0 ± 0.05	0	0	0	0 ± 0.16	0	0 ± 0.0004	0	0	0
CGG	0 ± 0.06	0	36 ± 6	0 ± 0.2	0 ± 0.03	0 ± 0.05	0	0	0	0	0	0	0
CGA	0	0 ± 0.02	0 ± 0.3	36 ± 6	0 ± 0.01	0 ± 0.02	0	0	0	0	0	0	0
CGT	0	0	0 ± 0.04	0 ± 0.01	36 ± 6	0 ± 0.3	0	0	0	0	0	0	0
CGC	0	0	0 ± 0.03	0 ± 0.02	0 ± 0.3	36 ± 6	0	0	0	0	0	0	0
AAG	0 ± 0.5	0	0	0	0	0	29 ± 6	0 ± 0.2	0 ± 0.004	0	0	0	0 ± 0.003
AAA	0	0 ± 0.5	0	0	0	0	0 ± 0.2	29 ± 6	0	0 ± 0.006	0	0	0
ACG	0 ± 0.09	0	0	0	0	0	0 ± 0.08	0	4 ± 3	0 ± 0.03	0 ± 0.02	0 ± 0.002	0 ± 0.006
ACA	0	0 ± 0.09	0	0	0	0	0	0 ± 0.02	0 ± 0.03	4 ± 3	0 ± 0.005	0 ± 0.004	0
ACT	0	0	0	0	0	0	0	0	0 ± 0.004	0 ± 0.006	4 ± 3	0 ± 0.03	0
ACC	0	0	0	0	0	0	0	0	0 ± 0.01	0 ± 0.007	0 ± 0.04	4 ± 3	0
ATG	0	0	0	0	0	0	0	0	0	0	0	0	0 ± 0.04
