# Supplementary material for: Mutational pathway maps and founder effects define the within-host spectrum of hepatitis C virus mutants resistant to drugs
Source: PLoS Pathog. 2019 Apr 1;15(4):e1007701. doi: 10.1371/journal.ppat.1007701 (PMC6459561; doi:10.1371/journal.ppat.1007701)
Supplement: S1 Text — (PDF) [file ppat.1007701.s012.pdf]

S1 Text. Estimation of unknown parameters

In the intracellular model, the parameters k_+, k_-, and K were unknown. We chose them to ensure that our simulations were consistent with the known overall viral replication dynamics in infected cells. Thus, we let the total RNA and RC populations to be 200 and 40 copies, respectively, at steady state [25], and let the steady state be reached in 48 h of infection [24]. In a deterministic framework, the populations would evolve according to the equations:

$$\frac{dRNA}{dt} = k_-\left(1 - \frac{RNA + RC}{K}\right)RC - k_+\left(1 - \frac{RNA + RC}{K}\right)RNA - \rho RNA - d_{RNA}RNA$$

$$\frac{dRC}{dt} = k_+\left(1 - \frac{RNA + RC}{K}\right)RNA - d_{RC}RC$$

which at steady state would yield $k_+ = \frac{40d_{RC}}{200\left(1 - \frac{240}{K}\right)}$ and $k_- = \frac{200(d_{RNA}+\rho)+40d_{RC}}{40\left(1-\frac{240}{K}\right)}$. Using the latter relationships and the estimates of the other parameters from Table 1, we solved the above equations stochastically for different values of K and found that K=270 yielded the appropriate dynamics (S9 Fig.). We therefore used this value and the corresponding values of k_+ and k_- obtained from the above relationships in our simulations.
